# Investigating sex-specific associations of lipid traits with type 2 diabetes, glycemic traits and sex hormones using Mendelian randomization

**DOI:** 10.1186/s12933-022-01714-2

**Published:** 2023-01-09

**Authors:** Guoyi Yang, C. Mary Schooling

**Affiliations:** 1 School of Public Health, Li Ka Shing Faculty of Medicine, The University of Hong Kong, Hong Kong, China; 2 Graduate School of Public Health and Health Policy, City University of New York, New York, USA

**Keywords:** Lipids, Mendelian randomization, Sex hormone, Type 2 diabetes

## Abstract

**Background:**

Low-density lipoprotein **(**LDL)-cholesterol is positively associated with cardiovascular disease (CVD) and inversely associated with type 2 diabetes, which could detract from lipid modification. Here, we examined whether lipid traits potentially relevant to CVD aetiology, i.e. apolipoprotein B (apoB), triglycerides (TG) and lipoprotein(a) [Lp(a)] exhibited the same associations. We investigated sex-specifically, including the role of sex hormones, because sex disparities exist in lipid profile and type 2 diabetes. We also replicated where possible.

**Methods:**

We used Mendelian randomization (MR) to examine sex-specific associations of apoB, TG and Lp(a) with type 2 diabetes, HbA1c, fasting insulin, fasting glucose, testosterone and estradiol in the largest relevant sex-specific genome-wide association studies (GWAS) in people of European ancestry and replicated where possible. We also assessed sex-specific associations of liability to type 2 diabetes with apoB, TG and Lp(a).

**Results:**

Genetically predicted apoB and Lp(a) had little association with type 2 diabetes or glycemic traits in women or men. Genetically predicted higher TG was associated with higher type 2 diabetes risk [odds ratio (OR) 1.44 per standard deviation (SD), 95% confidence interval (CI) 1.26 to 1.65], HbA1c and fasting insulin specifically in women. Higher TG was associated with lower testosterone in women and higher testosterone in men, but with lower estradiol in men and women. Genetic liability to type 2 diabetes was associated with higher TG in women, and possibly with lower apoB in men.

**Conclusions:**

Lipid traits potentially relevant to CVD aetiology do not exhibit contrasting associations with CVD and type 2 diabetes. However, higher TG is associated with higher type 2 diabetes risk and glycemic traits, which in turn further increases TG specifically in women, possibly driven by sex hormones.

**Supplementary Information:**

The online version contains supplementary material available at 10.1186/s12933-022-01714-2.

## Introduction

Low-density lipoprotein (LDL)-cholesterol is associated with higher risk of cardiovascular disease (CVD) and lower risk of type 2 diabetes [1, 2]. Similar associations have been observed for a major lipid modifier, statins [3], and for familial hypercholesteremia [4], indicating either lipid traits are overlooked causes of type 2 diabetes or additional underlying factors with opposing effects on CVD and type 2 diabetes exist, as previously suggested [5]. Understanding whether other lipids potentially relevant to the aetiology of CVD have opposing effects on CVD and type 2 diabetes has public health implications for clinical interventions and drug development for CVD prevention.

Apolipoprotein B (apoB) is emerging as the predominant trait in the aetiology of CVD, probably accounting for the effect of LDL-cholesterol on CVD and lifespan [6, 7]. The causal roles of triglycerides (TG) [8] and lipoprotein(a) [Lp(a)] [9] in CVD are gaining acceptance, whilst the roles of high-density lipoprotein (HDL)-cholesterol and correspondingly apolipoprotein A (apoA) are in doubt given drugs targeting HDL-cholesterol do not reduce CVD risk in trials [10, 11]. However, the association of apoB with type 2 diabetes risk remains unclear [7]. Previous studies investigating the associations of TG and Lp(a) with type 2 diabetes risk have yielded contradictory results, suggesting inverse [1, 12], null [13–15] or positive [2, 7, 16] associations of TG, and inverse [17] or null [18] associations of Lp(a).

Women usually have a less atherogenic lipid profiles than men [19] and lipids have differing associations with CVD by sex [20], while diabetes incidence is similar in men and women although differences by sex exist in pathophysiology and complications of type 2 diabetes, possibly partly driven by sex hormones [21, 22]. Estrogen protects against type 2 diabetes in women [23], while testosterone protects against type 2 diabetes in men [24]. Few studies have evaluated sex-specific associations of lipid traits with type 2 diabetes risk [16]. Randomized controlled trials (RCTs) are not usually powered to detect sex differences. Given the correlation between lipid fractions, it is also difficult to disentangle the role of each lipid fraction in the development of type 2 diabetes in an RCT.

To examine sex-specific associations of lipid traits potentially relevant to the aetiology of CVD, i.e. apoB, TG and Lp(a), with type 2 diabetes risk, glycemic traits and sex hormones, we conducted a Mendelian randomization (MR) study, i.e., an instrumental variable analysis with genetic instruments [25]. MR studies take advantage of genetic randomization at conception to obtain less confounded estimates [25]. We used multivariable MR to assess the robustness of the findings [26], and replicated, where possible, i.e. in East Asians.

## Methods

### Study design

We performed a two-sample MR study to examine sex-specific associations of apoB, TG and Lp(a) with type 2 diabetes, glycemic traits and sex hormones, taking advantage of the largest relevant publicly available sex-specific genetic summary statistics. We extracted sex-specific independent (r^2^ < 0.001) genome-wide significant (*p* value < 5 × 10^–8^) genetic instruments for each lipid trait from the UK Biobank (http://www.nealelab.is/uk-biobank/), and where possible, ancestry-specific genetic instruments from the Global Lipids Genetics Consortium (GLGC) excluding the UK Biobank participants [27] for replication. We applied them to the largest sex-specific genome-wide association study (GWAS) of type 2 diabetes, with different participants from the UK Biobank, in people of European ancestry [28], and then replicated in East Asians [29]. To give greatly granularity, we also applied these genetic instruments to the largest relevant sex-specific GWAS of HbA1c, fasting insulin, fasting glucose, testosterone and estradiol in people of European ancestry [30–32]. We used multivariable MR to assess the role of each lipid trait taking into account the others and additionally body mass index (BMI) [26], because BMI affects both lipid traits and type 2 diabetes [33], and might confound their associations given genetic instruments for lipid traits may also predict BMI. We also assessed sex-specific associations of genetic liability to type 2 diabetes with lipid traits, because bidirectional relationships between lipids and glycemic traits have previously been suggested [34].

### Genetic predictors for lipid traits

We extracted sex-specific independent (r^2^ < 0.001) genome-wide significant (*p* value < 5 × 10^–8^) genetic instruments for apoB (184,377 women/158,213 men), TG (184,885 women/159,107 men) and Lp(a) (147,684 women/126,212 men) from sex-specific GWAS of the UK Biobank (http://www.nealelab.is/uk-biobank). The quality controlled GWAS included people of white British ancestry, intended age 40–69 years, adjusted for age, age^2^, and the first 20 principal components. We also extracted ancestry-specific independent (r^2^ < 0.001) genome-wide significant (*p* value < 5 × 10^–8^) genetic instruments for TG (864,240/83,965 people of European/East Asian ancestry) from GLGC excluding the UK Biobank participants [27] for replication. Summary statistics were adjusted for age, age^2^, sex, principal components of ancestry and study-specific covariates [27].We excluded genetic variants located on the *GCKR* or *FADS1* genes as previously [13, 14, 16], because they are strongly associated with other traits relevant to type 2 diabetes.

To assess the validity of sex-specific genetic instruments from the UK Biobank in East Asians, we used coronary artery disease (CAD) (cases = 7708 women/21,611 men, controls = 95,398 women/87,736 men) from Biobank Japan [35] as a positive control outcome. Summary statistics were adjusted for age and top five principal components [35].

We conducted multivariable MR using sex-specific genetic instruments from the UK Biobank to identify the direct effect of each lipid, i.e. apoB, TG and Lp(a), accounting for potential confounding or mediation by the other lipids considered [26]. As the effects of lipid traits on type 2 diabetes might also be confounded by BMI [33], we additionally included BMI in the multivariable MR analyses. We obtained sex-specific genetic associations with BMI (193,570 women/166,413 men) from a GWAS of the UK Biobank (http://www.nealelab.is/uk-biobank), adjusted for age, age^2^, and the first 20 principal components. In multivariable MR, we combined all the genetic instruments, dropped duplicated SNPs and removed correlated (r^2^ ≥ 0.001) SNPs based on the minimum *p* value for genetic association with each trait. We extracted associations of the remaining SNPs with the exposures and the outcome, then fitted multivariable models.

### Genetic associations with type 2 diabetes

We obtained sex-specific genetic associations with type 2 diabetes from the DIAbetes Meta-ANalysis of Trans-Ethnic association studies (DIAMANTE) consortium in people of European ancestry excluding UK Biobank participants (cases = 23,197 women/29,583 men, controls = 201,329 women/193,076 men) [28]. Summary genetic associations were adjusted for study-specific covariates and principal components [28]. We extracted sex-specific independent (r^2^ < 0.001) genome-wide significant (*p* value < 5 × 10^–8^) genetic instruments for liability to type 2 diabetes from the GWAS [28].

We also obtained sex-specific associations with type 2 diabetes (cases = 27,370 women/28,027 men, controls = 135,055 women/89,312 men) from the Asian Genetic Epidemiology Network (AGEN) meta-analysis in East Asians, adjusted for age, sex, study-specific covariates and principal components of ancestry [29].

### Genetic associations with HbA1c, fasting insulin and fasting glucose

We obtained sex-specific genetic associations with HbA1c from a GWAS of the UK Biobank (185,022 women/159,160 men), adjusted for age, age^2^ and 20 principal components (http://www.nealelab.is/uk-biobank/). We obtained sex-specific genetic associations with fasting insulin (50,404 women/47,806 men) and fasting glucose (73,089 women/67,506 men) from the Meta-Analyses of Glucose and Insulin-related traits Consortium (MAGIC) in people of European ancestry without diabetes, which were adjusted for age, study site and principal components [30].

### Genetic associations with testosterone and estradiol

We obtained genetic associations with total testosterone in women (*N* = 230,454) and bioavailable testosterone, hereafter testosterone in men (*N* = 178,782), from a GWAS conducted in the UK Biobank, because they have little correlation with sex hormone-binding globulin (SHBG) [31]. Estimates were adjusted for genotyping chip/release of genetic data, age at baseline, fasting time, center and 10 genetically derived principal components [31].

We obtained sex-specific genetic associations with estradiol (above detection limit = 37,461 women/13,367 men, below detection limit = 126,524 women/134,323 men) from a GWAS of the UK Biobank [32]. Summary quality controlled genetic associations were adjusted for age, BMI, the first 10 genetic principal components, genotyping array, and additionally hormone replacement therapy, oral contraceptive use, number of live births, menopausal status, and hysterectomy in women [32].

### Statistical analysis

We used the F-statistic to assess instrument strength, obtained from the mean of the square of each SNP-exposure association divided by the square of its standard error [36]. An F-statistic larger than 10 suggests weak instrument bias is unlikely. In multivariable MR, we used the conditional F-statistic *F*_*TS*_ to examine the instrument strength for each exposure conditional on the other exposures, and the Q-statistic to assess heterogeneity [37].

We aligned the SNPs based on alleles and/or allele frequency and excluded palindromic SNPs with intermediate effect allele frequency (i.e. 0.42–0.58) when the strand direction was uncertain. We used proxy SNPs (r^2^ ≥ 0.8), where possible, when SNPs were not in the outcome GWAS. We obtained MR estimates by meta-analyzing Wald estimates (i.e. genetic association with the outcome divided by genetic association with the exposure) using inverse variance weighting (IVW) with first-order weights, and fixed effects for three SNPs or less and random effects for four SNPs or more [38]. IVW assumes all the genetic variants are valid or have balanced pleiotropy [38]. IVW using first-order weights gives unbiased estimates with or without the presence of heterogeneity, when the mean F-statistic is high [39]. To assess the validity of the IVW estimates, we conducted sensitivity analyses using methods with different assumptions, i.e. the weighted median [40], MR Egger [41] and the contamination mixture method [42]. The weighted median is valid when more than half of the information derives from valid SNPs [40]. MR Egger assumes no consequence of the instruments confounds exposure on outcome [41]. We used the MR Egger intercept to assess whether the IVW estimate might be affected by violation of the exclusion-restriction assumption [41]. The contamination mixture method is robust to outliers and horizontal pleiotropy, with well-controlled type 1 error rates [42, 43]. We used multivariable IVW to assess the direct effect of each lipid trait controlling for the others. We used multivariable MR Egger and additional adjustment for BMI as sensitivity analysis.

We used Steiger filtering to identify SNPs explaining more of the variance in the outcome than in the exposure (*p* value < 0.05) [44], given potentially bidirectional relationships between lipids and glycemic traits [34]. To assess whether MR estimates were affected by reverse causality, we conducted analyses including and excluding these SNPs.

Power was estimated based on the approximation that the sample size required for an MR study is the sample size for the conventional observational study divided by the variance in the exposure explained by the SNPs [45]. This variance was estimated as the sum of 2*beta^2^*MAF*(1-MAF), where beta is the standardized genetic association with the exposure and MAF is the minor allele frequency.

Sex differences were assessed using a two-sided z-test. A Bonferroni corrected significance level was set at α = 0.05/3 = 0.017, because 3 lipid fractions were included. All statistical analyses were conducted using R version 4.1.1 and the packages “TwoSampleMR” for harmonizing data, “MendelianRandomization” for univariable and multivariable MR, “MVMR” for conditional F-statistics and Q-statistics, and “ieugwasr” for removing correlated SNPs. All analyses were based on publicly available summary statistics, which does not require ethical approval.

## Results

### Genetic instruments

We extracted 111 (women) and 80 (men) independent (r^2^ < 0.001) genome-wide significant (*p* value < 5 × 10^–8^) SNPs for apoB, 144 (women) and 96 (men) for TG, and 15 (women) and 10 (men) for Lp(a) from the UK Biobank. All the SNPs had an F-statistic larger than 10. After excluding 2 genetic variants (rs780094 and rs1260326) located on *GCKR*, the mean F-statistics were 170.9 (women) and 188.9 (men) for apoB, 117.7 (women) and 139.9 (men) for TG, and 673.3 (women) and 1101.5 (men) for Lp(a). When instrumented by these SNPs, genetically predicted apoB, TG and Lp(a) were positively associated with the positive control outcome, i.e. CAD in East Asians, despite wide confidence intervals for Lp(a) (Additional file 1: Table S1).

We also extracted 238 (European) and 29 (East Asian) independent (r^2^ < 0.001) genome-wide significant (*p* value < 5 × 10^–8^) SNPs for TG from GLGC excluding the UK Biobank participants for replication. We excluded 2 SNPs (rs150419156 and rs1260326) on *GCKR* and 1 SNP (rs7394579) on *FADS1*. The F-statistics were all greater than 10, with mean 236.3 and 192.6 for people of European and East Asian ancestry, respectively.

In multivariable MR including apoB, TG and Lp(a), the conditional F-statistics were 68.1 (women) and 96.8 (men) for apoB, 69.6 (women) and 80.2 (men) for TG, and 46.8 (women) and 72.5 (men) for Lp(a). We extracted 148 (women) and 134 (men) independent (r^2^ < 0.001) genome-wide significant (*p* value < 5 × 10^–8^) SNPs for BMI, and additionally included BMI in the multivariable MR, when the conditional F-statistics were 43.4 (women) and 53.3 (men) for apoB, 49.3 (women) and 50.2 (men) for TG, 34.4 (women) and 45.7 (men) for Lp(a), and 21.7 (women) and 24.2 (men) for BMI.

We also extracted 33 (women) and 48 (men) independent (r^2^ < 0.001) genome-wide significant (*p* value < 5 × 10^–8^) genetic instruments for liability to type 2 diabetes from DIAMANTE in people of European ancestry excluding UK Biobank participants. The F-statistics were all greater than 10, with mean 63.5 and 68.3 for women and men, respectively.

Results of power calculations are shown in Additional file 1: Table S2.

### Sex-specific associations of lipid traits with type 2 diabetes and glycemic traits

**Fig. 1:**
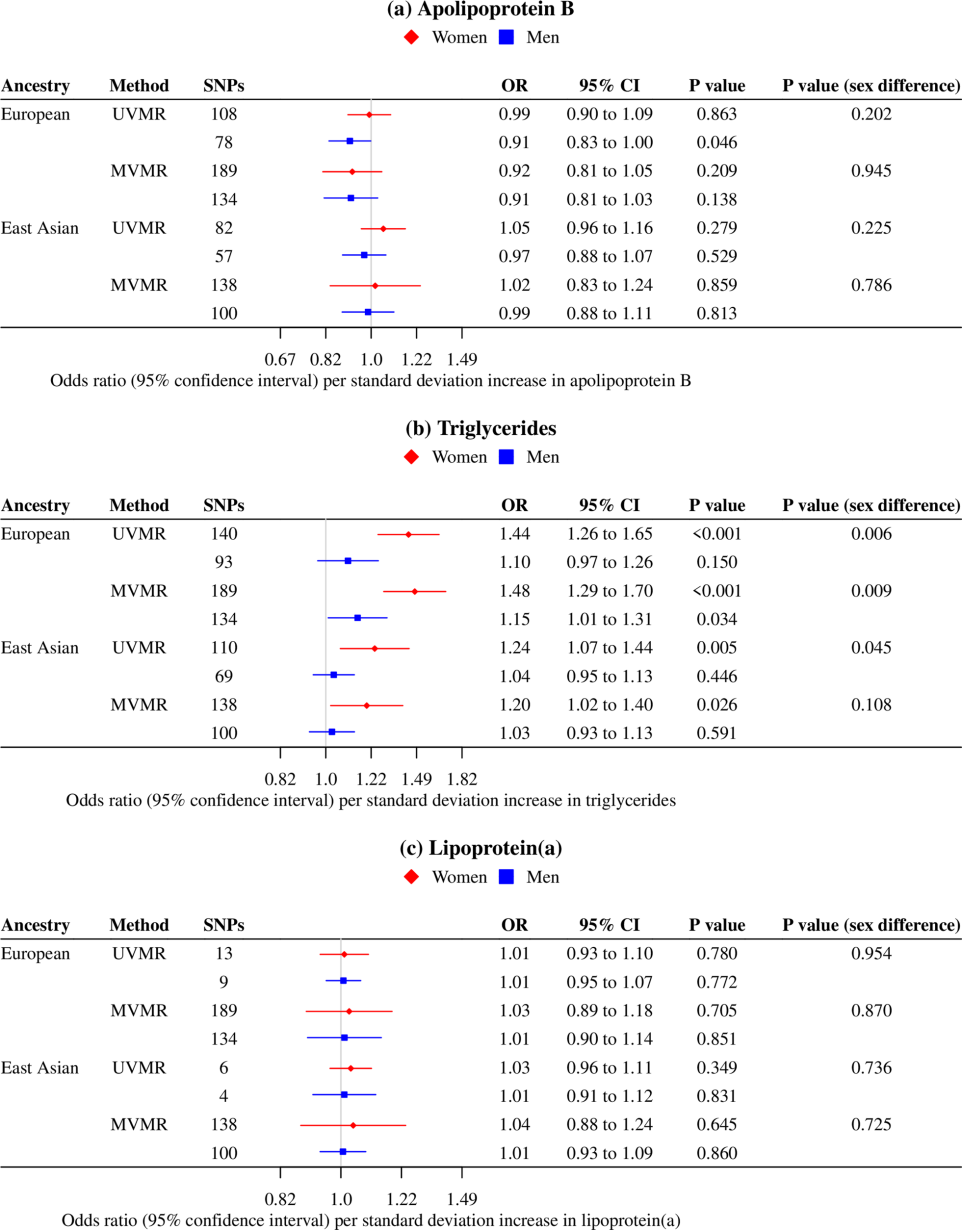
Ancestry- and sex-specific associations of genetically predicted lipid fractions (instrumented by the SNPs from the UK Biobank) with type 2 diabetes. **a**. *MVMR* multivariable MR, *UVMR* univariable MR, **b**. MVMR estimates for each lipid fraction were adjusted for the other two traits; **c**. Estimates were derived using inverse variance weighted approach, and were expressed in standard deviation for lipid fractions, and in odds ratio for type 2 diabetes

**Fig. 2:**
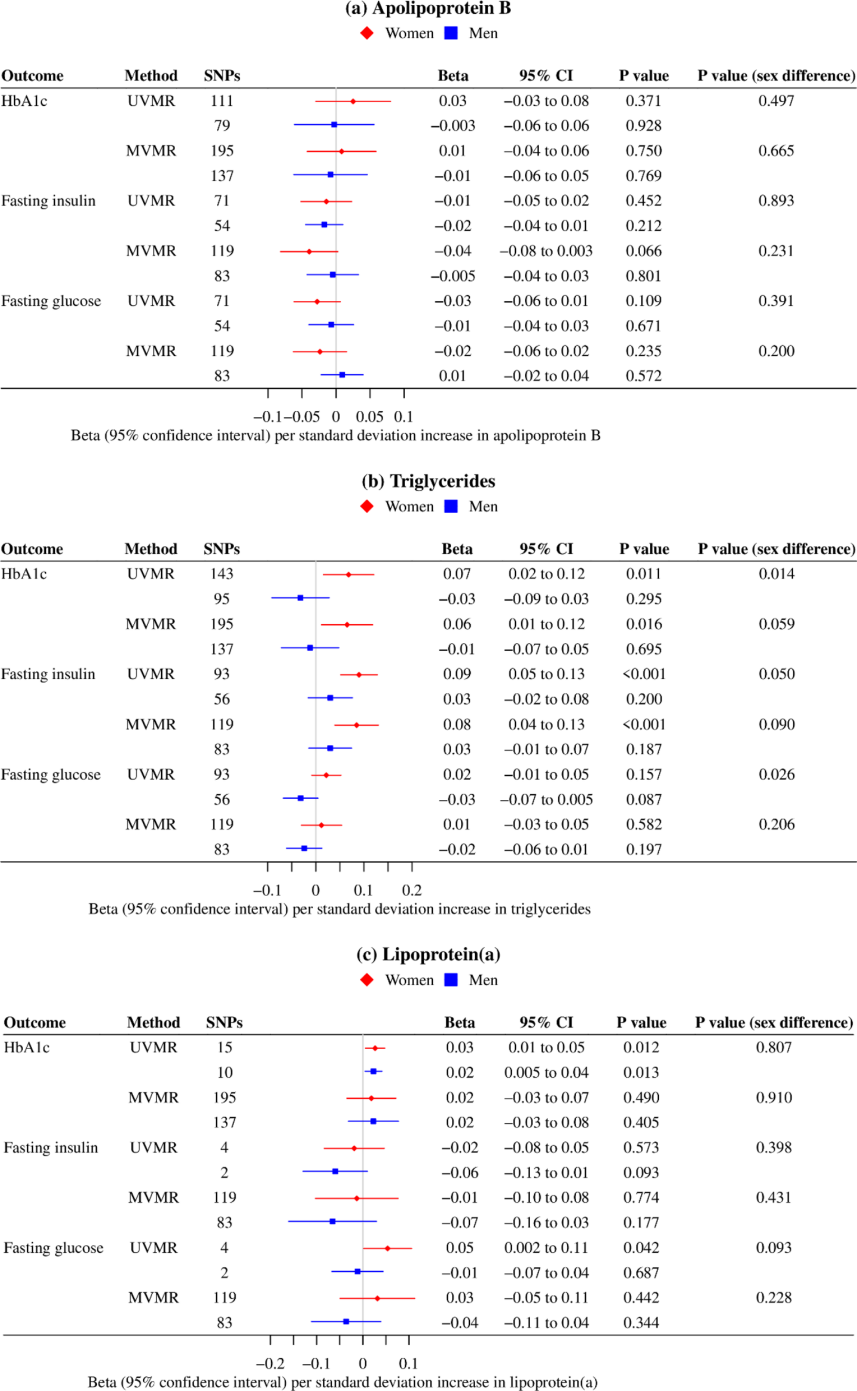
Sex-specific associations of genetically predicted lipid fractions (instrumented by the SNPs from the UK Biobank) with glycemic traits in people of European ancestry. **a**. *MVMR* multivariable MR, *UVMR* univariable MR, **b**. MVMR estimates for each lipid fraction were adjusted for the other two traits; **c**. Estimates were derived using inverse variance weighted approach, and were expressed in standard deviation for lipid fractions and HbA1c, in pmol/L (natural log transformed) for fasting insulin, and in mmol/L for fasting glucose

In the univariable MR analyses, genetically predicted apoB was not associated with type 2 diabetes risk or with any glycemic trait in women or men (Figs. 1, 2). Genetically predicted higher TG was associated with higher type 2 diabetes risk, HbA1c and fasting insulin in women, but not men (Figs. 1, 2). Findings were similar when using ancestry-specific TG SNPs from GLGC excluding the UK Biobank participants, although the MR Egger intercept indicated possible pleiotropy (Additional file 1: Table S3). Genetically predicted Lp(a) was not associated with type 2 diabetes risk in women or men (Fig. 1), despite a positive association with HbA1c (Fig. 2).

In the multivariable MR analyses, the associations of apoB with type 2 diabetes and glycemic traits were similar when taking into account TG and Lp(a) (Figs. 1, 2). The positive association of TG with type 2 diabetes became pronounced in men of European ancestry after controlling for apoB and Lp(a) (Fig. 1); however, the *p* value did not reach Bonferroni corrected significance. The positive association of Lp(a) with HbA1c was attenuated towards the null after controlling for apoB and TG (Fig. 2). These associations were robust to different analytic methods, additional adjustment for BMI and exclusion of SNPs explaining more of the variance in the outcome than in the exposure (Additional file 1: Table S4, S5, S6, S7). The associations of lipid traits with type 2 diabetes were replicated in East Asians (Fig. 1).

### Sex-specific associations of lipid traits with testosterone and estradiol

**Fig. 3:**
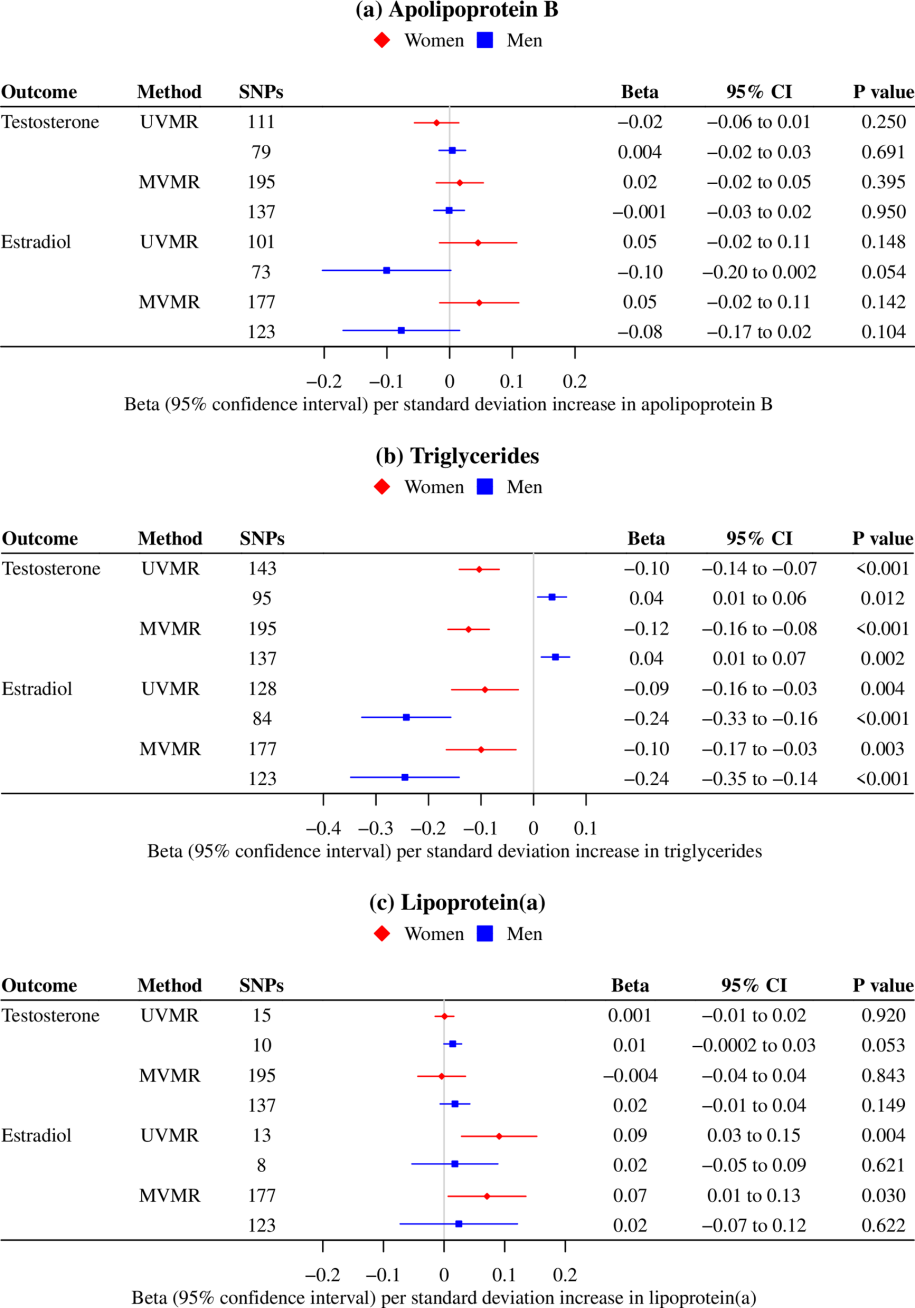
Sex-specific associations of genetically predicted lipid fractions (instrumented by the SNPs from the UK Biobank) with sex hormones in people of European ancestry. **a**. *MVMR* multivariable MR, *UVMR* univariable MR, **b**. MVMR estimates for each lipid fraction were adjusted for the other traits; **c**. Estimates were derived using inverse variance weighted approach, and were expressed in standard deviation for lipid fractions and testosterone, and in log odds for estradiol (above and below the limit of detection)

In the univariable MR analyses, genetically predicted apoB had little association with sex hormones in women or men (Fig. 3). Genetically predicted higher TG was associated with lower testosterone in women and higher testosterone in men, but with lower estradiol in men and women (Fig. 3). Findings were similar when instrumented by TG SNPs from GLGC excluding the UK Biobank (Additional file 1: Table S3). Genetically predicted higher Lp(a) was possibly associated with higher estradiol in women, but not in men (Fig. 3). Multivariable MR analyses taking into account the effects of other lipid traits gave consistent results (Fig. 3).

The MR Egger intercept indicated possible pleiotropy, and the Q-statistics suggested heterogeneity in multivariable MR (Additional file 1: Table S8). Nevertheless, findings were similar when using different analytic methods, additionally adjusting for BMI and excluding SNPs explaining more of the variance in the outcome than in the exposure (Additional file 1: Table S8, S9).

### Sex-specific associations of liability to type 2 diabetes with lipid traits

Genetic liability to type 2 diabetes was associated with higher TG in women, and possibly with lower apoB in men (Table 1, *p* values for sex difference 0.002 and 0.067, respectively). Findings were similar when using different analytic methods, and the MR Egger intercept did not indicate possible pleiotropy (Table 1). The inverse association of liability to type 2 diabetes with apoB became pronounced in men after excluding SNPs explaining more of the variance in the outcome than in the exposure (Table 1, *p* values for sex difference 0.001).

**Table 1:** Mendelian randomization estimates for sex-specific associations of genetic liability to type 2 diabetes with lipid fractions in people of European ancestry

Sex	Outcome	Method	Including SNPs identified by Steiger filtering					Excluding SNPs identified by Steiger filtering				
			SNPs	Beta	95% CI	*P* value	*P* value (intercept)	SNPs	Beta	95% CI	*P* value	*P* value (intercept)
Women	ApoB	IVW	33	0.02	− 0.02 to 0.05	0.311		32	0.02	− 0.00 to 0.05	0.089	
Women	ApoB	Weighted median	33	− 0.01	− 0.03 to 0.02	0.588		32	− 0.004	− 0.03 to 0.02	0.694	
Women	ApoB	MR Egger	33	0.03	− 0.06 to 0.11	0.570	0.846	32	0.01	− 0.07 to 0.08	0.863	0.601
Women	ApoB	Conmix	33	− 0.02	− 0.03 to 0.04	0.511		32	− 0.02	− 0.03 to 0.04	0.101	
Men	ApoB	IVW	48	− 0.06	− 0.14 to 0.02	0.120		47	− 0.03	− 0.05 to -0.01	< 0.001	
Men	ApoB	Weighted median	48	− 0.04	− 0.05 to -0.02	< 0.001		47	− 0.04	− 0.05 to -0.02	< 0.001	
Men	ApoB	MR Egger	48	− 0.04	− 0.22 to 0.14	0.641	0.833	47	− 0.04	− 0.07 to 0.00	0.068	0.752
Men	ApoB	Conmix	48	− 0.02	− 0.04 to -0.00	0.028		47	− 0.05	− 0.06 to -0.02	0.004	
Women	TG	IVW	33	0.13	0.08 to 0.17	< 0.001		29	0.09	0.07 to 0.12	< 0.001	
Women	TG	Weighted median	33	0.09	0.07 to 0.12	< 0.001		29	0.09	0.06 to 0.11	< 0.001	
Women	TG	MR Egger	33	0.05	− 0.06 to 0.16	0.346	0.145	29	0.07	0.02 to 0.13	0.014	0.462
Women	TG	Conmix	33	0.11	0.06 to 0.12	< 0.001		29	0.12	0.10 to 0.14	< 0.001	
Men	TG	IVW	48	0.03	− 0.01 to 0.07	0.104		46	0.03	0.01 to 0.06	0.014	
Men	TG	Weighted median	48	0.02	− 0.00 to 0.04	0.052		46	0.02	− 0.00 to 0.04	0.068	
Men	TG	MR Egger	48	0.06	− 0.03 to 0.16	0.200	0.512	46	0.06	− 0.00 to 0.12	0.065	0.385
Men	TG	Conmix	48	0.01	− 0.01 to 0.04	0.392		46	0.03	− 0.01 to 0.05	0.107	
Women	Lp(a)	IVW	33	− 0.01	− 0.03 to 0.00	0.067		33	− 0.01	− 0.03 to 0.00	0.067	
Women	Lp(a)	Weighted median	33	− 0.01	− 0.03 to 0.01	0.342		33	− 0.01	− 0.03 to 0.01	0.342	
Women	Lp(a)	MR Egger	33	0.004	− 0.03 to 0.04	0.831	0.313	33	0.004	− 0.03 to 0.04	0.831	0.313
Women	Lp(a)	Conmix	33	− 0.02	− 0.04 to 0.00	0.098		33	− 0.02	− 0.04 to 0.00	0.098	
Men	Lp(a)	IVW	48	− 0.01	− 0.02 to 0.01	0.319		48	− 0.01	− 0.02 to 0.01	0.319	
Men	Lp(a)	Weighted median	48	− 0.01	− 0.03 to 0.01	0.142		48	− 0.01	− 0.03 to 0.01	0.142	
Men	Lp(a)	MR Egger	48	− 0.03	− 0.06 to 0.00	0.076	0.138	48	− 0.03	− 0.06 to 0.00	0.076	0.138
Men	Lp(a)	Conmix	48	− 0.01	− 0.02 to -0.00	0.097		48	− 0.01	− 0.02 to -0.00	0.097	

The SNPs explaining more of the variance in the outcome than in liability to type 2 diabetes identified by Steiger filtering are rs66787104 for apoB in women, rs429358 for apoB in men, rs13391980, rs1399625, rs1562397 and rs2925979 for TG in women, and rs2943657 and rs429358 for TG in men. Estimates are expressed in standard deviation for lipid fractions, and in log odds for type 2 diabetes

*ApoB* apolipoprotein B, *CI* confidence interval, *Conmix* contamination mixture method, *IVW* inverse variance weighted, *Lp(a)* lipoprotein(a), *TG* triglycerides

## Discussion

Consistent with previous studies [2, 7, 16, 18], we found a positive association of TG with type 2 diabetes but little association of Lp(a) with type 2 diabetes. We added by showing apoB was not associated with type 2 diabetes or glycemic traits, but TG was positively associated with type 2 diabetes and glycemic traits specifically in women. The finding that lipid traits potentially relevant to the aetiology of CVD do not exhibit contrasting associations with risk of CVD and type 2 diabetes is novel and has implications for interventions and drug development for CVD prevention.

We found apoB, the predominant trait in the aetiology of CVD [6, 46], was not associated with type 2 diabetes or any glycemic trait, in contrast to previous MR studies showing an inverse association of LDL-cholesterol with type 2 diabetes [1, 2]. Despite the high correlation between apoB and LDL-cholesterol, the mass of cholesterol per apoB particle is not uniform [47]. For a given value of apoB, the level of LDL-cholesterol increases when apoB particles are cholesterol-enriched [47]. In this situation, the uptake of cholesterol via the LDL receptor reduces, which might improve beta cell function and protect against type 2 diabetes [48]. Although diabetogenic effects of lipid modifiers have been observed, these effects are drug-specific rather than a general property of lipid lowering. Meta-analysis of RCTs suggest that only statins, of lipid modifiers currently in use, increase the risk of incident diabetes [49], possibly due to underlying factors with opposite effects on CVD and type 2 diabetes, such as sex hormones [5].

Our finding that higher TG was associated with higher type 2 diabetes risk and glycemic traits specifically in women is inconsistent with some previous MR studies showing inverse [1, 12] or null [13–15] associations of TG with type 2 diabetes. However, these studies used fewer SNPs [13–15], had smaller sample sizes [12–15], did not use analytic methods robust to pleiotropy [1, 12], and did not assess sex-specific associations [1, 12–15]. Our finding is consistent with an RCT showing bezafibrate lowering TG decreases type 2 diabetes incidence [50]. Differences by sex are consistent with observational studies showing a stronger relation of TG with diabetes in women than men [51, 52], but inconsistent with the Tehran Lipid and Glucose Study suggesting women experience more adverse changes in BMI and TG than men before the onset of diabetes [53]. However, this observation is possibly due to higher insulin sensitivity in women [21], and thus they may experience a greater burden of metabolic risk factors than men before diabetes becomes evident. Notably, the causal relation of BMI with type 2 diabetes is also stronger in women than men [54].

Genetically predicted TG had an inverse association with estradiol, and had sex-specific associations with testosterone, which may underlie sex-specific associations of TG with type 2 diabetes and glycemic traits. Estrogen protects against type 2 diabetes in women in trials [23], mainly through activating estrogen receptor α in various tissues (e.g. brain, liver, skeletal muscle, adipose tissue and pancreatic beta cells) and thereby improving adiposity, insulin sensitivity and glucose tolerance [21]. TG lowering estradiol in women could increase the risk of type 2 diabetes specifically in women. Consistently, higher TG is also associated with lower breast cancer risk [55] and lower bone mineral density [56]. Testosterone protects against type 2 diabetes in men [24], possibly due to improvement in body composition and insulin sensitivity [57]. Thus, TG increasing testosterone in men may ameliorate any effects of TG on type 2 diabetes. TG may also work via altering insulin-like growth factor-1 (IGF-1) and increase type 2 diabetes risk [58, 59]. However, the causal association of IGF-1 with type 2 diabetes is complex and differs by underlying molecular pathway [60]. Furthermore, sex disparities in the treatment of dyslipidemia could contribute to the positive association of TG with type 2 diabetes in women [61], but would not explain why the difference is specific to TG.

Consistent with a previous MR study suggesting bidirectional relationships between lipids and glycemic traits [34], we add by showing these relationships may differ by sex. Specifically, higher TG was associated with higher type 2 diabetes risk and glycemic traits, which in turn would increase TG in women. It has clinical implications that women might benefit more from early control of TG than men. Observationally, the relative risk of CVD associated with TG is higher in women than men [62], though not consistently so [63]. Further studies are warranted to assess whether the associations of TG with CVD and lifespan differ by sex, and to investigate the role of sex hormones in mediating these associations.

This is the first MR study comprehensively assessing sex-specific associations of lipid traits relevant to the aetiology of CVD with type 2 diabetes and glycemic traits, in both people of European and East Asian ancestry. Nevertheless, this study has several limitations. First, MR should fulfill three rigorous assumptions of relevance, independence and exclusion-restriction [25]. To fulfill the relevance assumption, we checked that F-statistics and conditional F-statistics were > 10, which suggests little weak instrument bias. To satisfy the independence assumption, we excluded the genetic variants located on the *GCKR* or *FADS1* genes as previously [13, 14, 16], because these genes are strongly associated with other traits relevant to type 2 diabetes. To address the exclusion restriction assumption, we used analytic methods with different assumptions and multivariable MR to control for potential pleiotropy via other lipid traits and BMI, which gave consistent conclusions. Second, we extracted genetic instruments for lipid traits from the same study (i.e. the UK Biobank) as we obtained the genetic effects on sex hormones. However, two-sample MR methods in a one-sample setting perform well within large biobanks, except for the MR Egger estimate, when the variability of instrument strength across variants (I^2^_GX_) is < 97% [64], which was not the case here. The bias due to overlapping sample is proportional to 1/F-statistic [65] and unlikely changes the results substantially given the high F-statistics (average F-statistics larger than 100 for all lipid traits in women and men). In addition, the associations of TG with sex hormones were replicated using the genetic instruments extracted from GLGC excluding the UK Biobank participants. Third, we extracted genetic instruments for lipid traits from a GWAS performed in people of European ancestry and used them to derive MR estimates in East Asians. However, using these SNPs gave expected results for CAD in East Asians. In addition, findings were similar when using TG SNPs obtained from East Asians. Fourth, MR, particularly for dichotomous outcomes, could be open to selection bias [66]. The associations of lipid traits with type 2 diabetes could be biased when the underlying sample is selected on surviving to recruitment on genetic make-up and competing risks of type 2 diabetes [66]. However, the participants were relatively young likely obviating selective survival to recruitment on genetic endowment. Furthermore, we obtained similar findings when using continuous outcomes, i.e. glycemic traits which are less likely affected by selection bias [66]. We obtained genetic associations of fasting insulin and fasting glucose from MAGIC only including individuals without diabetes [30], which may underestimate the associations. Fifth, the associations in people of European ancestry may not be transportable to other populations. However, causal effects should act consistently across settings, unless the mediating mechanisms differ [67]. We replicated the associations in East Asians; further investigation in other populations would be worthwhile. Sixth, we assessed whether MR estimates were affected by reverse causality using Steiger filtering, which calculated the variance explained in the exposure and the outcome by the genetic instruments [44]. However, Steiger filtering might have reduced statistical power or infer the wrong direction when measurement error differs between the exposure and the outcome [44]. Seventh, estradiol was taken as a binary phenotype (above and below the limit of detection) [32], and thus it is difficult to interpretate the magnitude of the estimates and compare the associations of lipid traits with estradiol between women and men. Finally, MR assesses lifetime effects of lipids, which could differ from the short-term effects of lipid-lowering drugs.

## Conclusions

Lipid traits potentially relevant to the aetiology of CVD do not exhibit contrasting associations with risk of CVD and type 2 diabetes. However, higher TG was positively associated with type 2 diabetes risk and glycemic traits, which in turn would increase TG specifically in women, possibly driven by sex hormones. These insights have implications for public health interventions and drug development for CVD prevention including identifying potential side-effects and reinforcing the importance of using sex-specific approaches in the investigation, prevention, treatment and management of CVD and type 2 diabetes.

## Supplementary Information

**Additional file 1: Table S1.** Mendelian Mendelian randomization estimates for sex-specific associations of genetically predicted lipid traits (instrumented by the SNPs from the UK Biobank) with coronary artery disease in East Asians. **Table S2.** Odds ratio or mean difference in z-score that was detectable at 80% power (α = 0.05) for each analysis. **Table S3.** Mendelian randomization estimates for ancestry- and sex-specific associations of genetically predicted triglycerides (instrumented by the SNPs from GLGC excluding the UK Biobank participants) with type 2 diabetes, glycemic traits and sex hormones. **Table S4.** Mendelian randomization estimates for ancestry- and sex-specific associations of genetically predicted lipid traits (instrumented by the SNPs from the UK Biobank) with type 2 diabetes (including SNPs explaining more of the variance in the outcome than in the exposure). **Table S5.** Mendelian randomization estimates for sex-specific associations of genetically predicted lipid traits (instrumented by the SNPs from the UK Biobank) with glycemic traits in people of European ancestry (including SNPs explaining more of the variance in the outcome than in the exposure). **Table S6.** Mendelian randomization estimates for ancestry- and sex-specific associations of genetically predicted lipid traits (instrumented by the SNPs from the UK Biobank) with type 2 diabetes (excluding SNPs explaining more of the variance in the outcome than in the exposure). **Table S7.** Mendelian randomization estimates for sex-specific associations of genetically predicted lipid traits (instrumented by the SNPs from the UK Biobank) with glycemic traits in people of European ancestry (excluding SNPs explaining more of the variance in the outcome than in the exposure). **Table S8.** Mendelian randomization estimates for sex-specific associations of genetically predicted lipid traits (instrumented by the SNPs from the UK Biobank) with sex hormones in people of European ancestry (including SNPs explaining more of the variance in the outcome than in the exposure). **Table S9.** Mendelian randomization estimates for sex-specific associations of genetically predicted lipid traits (instrumented by the SNPs from the UK Biobank) with sex hormones in people of European ancestry (excluding SNPs explaining more of the variance in the outcome than in the exposure).

**Additional file 2: Table S10.** Genetic variants for apolipoprotein B and their associations with type 2 diabetes, glycemic traits and sex hormones in women. **Table S11.** Genetic variants for apolipoprotein B and their associations with type 2 diabetes, glycemic traits and sex hormones in men. **Table S12.** Genetic variants for triglycerides and their associations with type 2 diabetes, glycemic traits and sex hormones in women. **Table S13.** Genetic variants for triglycerides and their associations with type 2 diabetes, glycemic traits and sex hormones in men. **Table S14.** Genetic variants for lipoprotein(a) and their associations with type 2 diabetes, glycemic traits and sex hormones in women. **Table S15.** Genetic variants for lipoprotein(a) and their associations with type 2 diabetes, glycemic traits and sex hormones in men. **Table S16.** Genetic variants for multivariable Mendelian randomization analyses and their associations with type 2 diabetes, glycemic traits and sex hormones in women. **Table S17.** Genetic variants for multivariable Mendelian randomization analyses and their associations with type 2 diabetes, glycemic traits and sex hormones in men.

## Data Availability

Summary statistics are available in the website http://www.nealelab.is/uk-biobank/ for UK Biobank (Neale lab), http://jenger.riken.jp/en/result for Biobank Japan, http://csg.sph.umich.edu/willer/public/glgc-lipids2021/ for GLGC, http://diagram-consortium.org/downloads.html for DIAMANTE, https://magicinvestigators.org/downloads/ for MAGIC, https://blog.nus.edu.sg/agen/summary-statistics/t2d-2020/ for AGEN, and https://www.ebi.ac.uk/gwas/ for GWAS of testosterone and estradiol. The data analyzed in the current study are provided in Additional file 2: Table S10, S11, S12, S13, S14, S15, S16, S17.

## Abbreviations

AGEN: Asian genetic epidemiology network
ApoA: Apolipoprotein A
ApoB: Apolipoprotein B
BMI: Body mass index
CI: Confidence interval
CVD: Cardiovascular diseases
DIAMANTE: DIAbetes Meta-ANalysis of Trans-Ethnic association studies
GLGC: Global lipids genetics consortium
GWAS: Genome-wide association studies
HDL: High-density lipoprotein
IGF-1: Insulin-like growth factor-1
IVW: Inverse variance weighting
LDL: Low-density lipoprotein
Lp(a): Lipoprotein(a)
MAGIC: Meta-analyses of glucose and insulin-related traits consortium
MR: Mendelian randomization
OR: Odds ratio
RCT: Randomized controlled trial
SD: Standard deviation
SHBG: Sex hormone-binding globulin
TG: Triglycerides
