# Supplementary material for: Investigating sex-specific associations of lipid traits with type 2 diabetes, glycemic traits and sex hormones using Mendelian randomization
Source: Cardiovasc Diabetol. 2023 Jan 9;22:3. doi: 10.1186/s12933-022-01714-2 (PMC9830908; doi:10.1186/s12933-022-01714-2)
Supplement: Supplementary file 1 — Additional file 1: Table S1. Mendelian Mendelian randomization estimates for sex-specific associations of genetically predicted lipid traits (instrumented by the SNPs from the UK Biobank) with coronary artery disease in East Asians. Table S2. Odds ratio or mean difference in z-score that was detectable at 80% power (α = 0.05) for each analysis. Table S3. Mendelian randomization estimates for ancestry- and sex-specific associations of genetically predicted triglycerides (instrumented by the SNPs from GLGC excluding the UK Biobank participants) with type 2 diabetes, glycemic traits and sex hormones. Table S4. Mendelian randomization estimates for ancestry- and sex-specific associations of genetically predicted lipid traits (instrumented by the SNPs from the UK Biobank) with type 2 diabetes (including SNPs explaining more of the variance in the outcome than in the exposure). Table S5. Mendelian randomization estimates for sex-specific associations of genetically predicted lipid traits (instrumented by the SNPs from the UK Biobank) with glycemic traits in people of European ancestry (including SNPs explaining more of the variance in the outcome than in the exposure). Table S6. Mendelian randomization estimates for ancestry- and sex-specific associations of genetically predicted lipid traits (instrumented by the SNPs from the UK Biobank) with type 2 diabetes (excluding SNPs explaining more of the variance in the outcome than in the exposure). Table S7. Mendelian randomization estimates for sex-specific associations of genetically predicted lipid traits (instrumented by the SNPs from the UK Biobank) with glycemic traits in people of European ancestry (excluding SNPs explaining more of the variance in the outcome than in the exposure). Table S8. Mendelian randomization estimates for sex-specific associations of genetically predicted lipid traits (instrumented by the SNPs from the UK Biobank) with sex hormones in people of European ancestry (including SNPs explaining more o [file 12933_2022_1714_MOESM1_ESM.pdf]

Additional file 1: Table S1. Mendelian randomization estimates for sex-specific associations of genetically predicted lipid traits (instrumented by the SNPs from the UK Biobank) with coronary artery disease in East Asians.

Sex	Exposure	Method	SNPs	OR	95% CI	*P* value	*P* value (Egger intercept)
Women	ApoB	IVW	74	1.16	1.02 to 1.33	0.025	
Women	ApoB	Weighted median	74	0.90	0.76 to 1.07	0.224	
Women	ApoB	MR Egger	74	1.04	0.84 to 1.28	0.736	0.164
Women	ApoB	Conmix	74	0.93	0.80 to 1.09	0.336	
Men	ApoB	IVW	57	1.40	1.17 to 1.67	<0.001	
Men	ApoB	Weighted median	57	1.39	1.25 to 1.54	<0.001	
Men	ApoB	MR Egger	57	1.49	1.17 to 1.89	0.001	0.479
Men	ApoB	Conmix	57	1.47	1.24 to 1.60	0.001	
Women	TG	IVW	101	1.28	1.13 to 1.44	<0.001	
Women	TG	Weighted median	101	1.25	1.08 to 1.45	0.003	
Women	TG	MR Egger	101	1.18	0.98 to 1.40	0.076	0.223
Women	TG	Conmix	101	1.27	1.13 to 1.39	<0.001	
Men	TG	IVW	63	1.12	1.02 to 1.23	0.022	
Men	TG	Weighted median	63	1.10	1.01 to 1.20	0.024	
Men	TG	MR Egger	63	1.07	0.94 to 1.23	0.298	0.423
Men	TG	Conmix	63	1.05	0.98 to 1.13	0.130	
Women	Lp(a)	IVW	5	1.11	0.94 to 1.31	0.211	
Women	Lp(a)	Weighted median	5	1.10	0.98 to 1.23	0.112	
Women	Lp(a)	MR Egger	5	1.08	0.78 to 1.50	0.646	0.833
Women	Lp(a)	Conmix	5	1.09	0.92 to 1.33	0.082	
Men	Lp(a)	IVW	3	1.04	0.98 to 1.10	0.162	
Men	Lp(a)	Weighted median	3	1.02	0.96 to 1.08	0.509	
Men	Lp(a)	MR Egger	3	0.80	0.31 to 2.08	0.645	0.560
Men	Lp(a)	Conmix	3	1.02	0.96 to 1.07	0.521	

a. ApoB, apolipoprotein B; CI, confidence interval; Conmix, contamination mixture method; IVW, inverse variance weighted; Lp(a), lipoprotein(a); OR, odds ratio; TG, triglycerides.

b. Estimates are expressed in standard deviation for lipid fractions, and in odds ratio for coronary artery disease.

Additional file 1: Table S2. Odds ratio or mean difference in z-score that was detectable at 80% power (α=0.05) for each analysis.

Ancestry	Sex	Exposure	Outcome	Measure	OR/mean difference in z-score detectable at 80% power (α=0.05)
European	Women	ApoB	Type 2 diabetes	OR	0.94
European	Men	ApoB	Type 2 diabetes	OR	0.94
East Asian	Women	ApoB	Type 2 diabetes	OR	0.94
East Asian	Men	ApoB	Type 2 diabetes	OR	0.94
European	Women	ApoB	HbA1c	Beta	0.02
European	Men	ApoB	HbA1c	Beta	0.02
European	Women	ApoB	Fasting insulin	Beta	0.04
European	Men	ApoB	Fasting insulin	Beta	0.04
European	Women	ApoB	Fasting glucose	Beta	0.03
European	Men	ApoB	Fasting glucose	Beta	0.04
European	Women	ApoB	Testosterone	Beta	0.02
European	Men	ApoB	Testosterone	Beta	0.02
European	Women	ApoB	Estradiol	OR	0.95
European	Men	ApoB	Estradiol	OR	0.92
European	Women	TG	Type 2 diabetes	OR	0.93
European	Men	TG	Type 2 diabetes	OR	0.94
East Asian	Women	TG	Type 2 diabetes	OR	0.94
East Asian	Men	TG	Type 2 diabetes	OR	0.94
European	Women	TG	HbA1c	Beta	0.02
European	Men	TG	HbA1c	Beta	0.02
European	Women	TG	Fasting insulin	Beta	0.04
European	Men	TG	Fasting insulin	Beta	0.04
European	Women	TG	Fasting glucose	Beta	0.04
European	Men	TG	Fasting glucose	Beta	0.04
European	Women	TG	Testosterone	Beta	0.02
European	Men	TG	Testosterone	Beta	0.02
European	Women	TG	Estradiol	OR	0.94
European	Men	TG	Estradiol	OR	0.91

European	Women	Lp(a)	Type 2 diabetes	OR	0.93
European	Men	Lp(a)	Type 2 diabetes	OR	0.94
East Asian	Women	Lp(a)	Type 2 diabetes	OR	0.93
East Asian	Men	Lp(a)	Type 2 diabetes	OR	0.94
European	Women	Lp(a)	HbA1c	Beta	0.02
European	Men	Lp(a)	HbA1c	Beta	0.02
European	Women	Lp(a)	Fasting insulin	Beta	0.05
European	Men	Lp(a)	Fasting insulin	Beta	0.04
European	Women	Lp(a)	Fasting glucose	Beta	0.04
European	Men	Lp(a)	Fasting glucose	Beta	0.04
European	Women	Lp(a)	Testosterone	Beta	0.02
European	Men	Lp(a)	Testosterone	Beta	0.02
European	Women	Lp(a)	Estradiol	OR	0.94
European	Men	Lp(a)	Estradiol	OR	0.92

a. ApoB, apolipoprotein B; Lp(a), lipoprotein(a); OR, odds ratio; TG, triglycerides.

Additional file 1: Table S3. Mendelian randomization estimates for ancestry- and sex-specific associations of genetically predicted triglycerides (instrumented by the SNPs from GLGC excluding the UK Biobank participants) with type 2 diabetes, glycemic traits and sex hormones.

Ancestry	Sex	Outcome	Method	SNPs	Measure	Estimate	95% CI	*P* value	*P* value (Egger intercept)
European	Women	Type 2 diabetes	IVW	229	OR	1.42	1.23 to 1.64	<0.001	
European	Women	Type 2 diabetes	Weighted median	229	OR	1.09	0.96 to 1.23	0.174	
European	Women	Type 2 diabetes	MR Egger	229	OR	0.83	0.68 to 1.01	0.057	<0.001
European	Women	Type 2 diabetes	Conmix	229	OR	1.20	1.08 to 1.33	0.001	
European	Men	Type 2 diabetes	IVW	229	OR	1.24	1.08 to 1.42	0.002	
European	Men	Type 2 diabetes	Weighted median	229	OR	1.00	0.88 to 1.13	0.938	
European	Men	Type 2 diabetes	MR Egger	229	OR	0.76	0.63 to 0.92	0.005	<0.001
European	Men	Type 2 diabetes	Conmix	229	OR	1.12	1.01 to 1.46	0.039	
East Asian	Women	Type 2 diabetes	IVW	27	OR	1.06	0.96 to 1.17	0.275	
East Asian	Women	Type 2 diabetes	Weighted median	27	OR	1.12	1.04 to 1.22	0.004	
East Asian	Women	Type 2 diabetes	MR Egger	27	OR	1.10	0.94 to 1.28	0.230	0.515
East Asian	Women	Type 2 diabetes	Conmix	27	OR	1.10	1.04 to 1.19	0.014	
East Asian	Men	Type 2 diabetes	IVW	27	OR	0.95	0.87 to 1.04	0.282	
East Asian	Men	Type 2 diabetes	Weighted median	27	OR	0.98	0.91 to 1.07	0.708	
East Asian	Men	Type 2 diabetes	MR Egger	27	OR	0.99	0.87 to 1.14	0.934	0.408
East Asian	Men	Type 2 diabetes	Conmix	27	OR	0.76	0.68 to 0.85	0.005	
European	Women	HbA1c	IVW	229	Beta	0.08	0.03 to 0.14	0.002	
European	Women	HbA1c	Weighted median	229	Beta	-0.03	-0.06 to 0.01	0.139	

European	Women	HbA1c	MR Egger	229	Beta	-0.09	-0.17 to -0.02	0.012	<0.001
European	Women	HbA1c	Conmix	229	Beta	0.35	0.25 to 0.41	0.009	
European	Men	HbA1c	IVW	229	Beta	0.01	-0.04 to 0.07	0.613	
European	Men	HbA1c	Weighted median	229	Beta	-0.08	-0.12 to -0.03	0.001	
European	Men	HbA1c	MR Egger	229	Beta	-0.19	-0.27 to -0.12	<0.001	<0.001
European	Men	HbA1c	Conmix	229	Beta	0.27	0.23 to 0.33	<0.001	
European	Women	Fasting insulin	IVW	187	Beta	0.07	0.03 to 0.12	0.001	
European	Women	Fasting insulin	Weighted median	187	Beta	0.01	-0.04 to 0.07	0.653	
European	Women	Fasting insulin	MR Egger	187	Beta	-0.05	-0.12 to 0.02	0.136	<0.001
European	Women	Fasting insulin	Conmix	187	Beta	0.03	-0.01 to 0.09	0.101	
European	Men	Fasting insulin	IVW	187	Beta	0.03	-0.02 to 0.07	0.208	
European	Men	Fasting insulin	Weighted median	187	Beta	-0.01	-0.07 to 0.05	0.749	
European	Men	Fasting insulin	MR Egger	187	Beta	-0.12	-0.19 to -0.06	<0.001	<0.001
European	Men	Fasting insulin	Conmix	187	Beta	0.10	0.04 to 0.17	0.005	
European	Women	Fasting glucose	IVW	187	Beta	0.00	-0.04 to 0.03	0.814	
European	Women	Fasting glucose	Weighted median	187	Beta	0.02	-0.03 to 0.06	0.453	
European	Women	Fasting glucose	MR Egger	187	Beta	-0.08	-0.14 to -0.03	0.002	<0.001
European	Women	Fasting glucose	Conmix	187	Beta	0.03	0.01 to 0.08	0.035	
European	Men	Fasting glucose	IVW	187	Beta	-0.02	-0.06 to 0.02	0.290	
European	Men	Fasting glucose	Weighted median	187	Beta	-0.02	-0.07 to 0.03	0.373	
European	Men	Fasting glucose	MR Egger	187	Beta	-0.10	-0.16 to -0.05	<0.001	<0.001
European	Men	Fasting glucose	Conmix	187	Beta	0.01	-0.03 to 0.06	0.892	

European	Women	Testosterone	IVW	230	Beta	-0.14	-0.18 to -0.10	<0.001	
European	Women	Testosterone	Weighted median	230	Beta	-0.07	-0.09 to -0.04	<0.001	
European	Women	Testosterone	MR Egger	230	Beta	-0.12	-0.17 to -0.06	<0.001	0.352
European	Women	Testosterone	Conmix	230	Beta	-0.06	-0.07 to -0.04	<0.001	
European	Men	Testosterone	IVW	230	Beta	0.02	-0.01 to 0.05	0.288	
European	Men	Testosterone	Weighted median	230	Beta	0.02	-0.01 to 0.05	0.187	
European	Men	Testosterone	MR Egger	230	Beta	0.03	-0.02 to 0.07	0.247	0.546
European	Men	Testosterone	Conmix	230	Beta	0.00	-0.04 to 0.04	0.804	
European	Women	Estradiol	IVW	209	logOR	-0.10	-0.17 to -0.03	0.004	
European	Women	Estradiol	Weighted median	209	logOR	-0.11	-0.21 to -0.00	0.048	
European	Women	Estradiol	MR Egger	209	logOR	-0.10	-0.20 to 0.01	0.062	0.954
European	Women	Estradiol	Conmix	209	logOR	-0.13	-0.19 to -0.06	0.003	
European	Men	Estradiol	IVW	209	logOR	-0.31	-0.40 to -0.23	<0.001	
European	Men	Estradiol	Weighted median	209	logOR	-0.32	-0.44 to -0.19	<0.001	
European	Men	Estradiol	MR Egger	209	logOR	-0.32	-0.45 to -0.19	<0.001	0.894
European	Men	Estradiol	Conmix	209	logOR	-0.37	-0.45 to -0.22	<0.001	

a. CI, confidence interval; Conmix, contamination mixture method; GLGC, Global Lipids Genetics Consortium; IVW, inverse variance weighted; OR, odds ratio.

b. Estimates are expressed in standard deviation for triglycerides, HbA1c and testosterone, in odds ratio for type 2 diabetes, in pmol/L (natural log transformed) for fasting insulin, in mmol/L for fasting glucose, and in log odds ratio (above and below the limit of detection) for estradiol.

Additional file 1: Table S4. Mendelian randomization estimates for ancestry- and sex-specific associations of genetically predicted lipid traits (instrumented by the SNPs from the UK Biobank) with type 2 diabetes (including SNPs explaining more of the variance in the outcome than in the exposure).

Ancestry	Sex	Exposure	Method	SNPs	OR	95% CI	*P* value	*P* value (Egger intercept)	*P* value (Q-statistics)
European	Women	ApoB	IVW	108	0.99	0.90 to 1.09	0.863		
European	Women	ApoB	Weighted median	108	0.91	0.83 to 1.00	0.063		
European	Women	ApoB	MR Egger	108	0.90	0.79 to 1.03	0.110	0.041	
European	Women	ApoB	Conmix	108	0.95	0.89 to 1.03	0.252		
European	Women	ApoB	MVMR1-IVW	189	0.92	0.81 to 1.05	0.209		
European	Women	ApoB	MVMR1-Egger	189	0.85	0.73 to 0.98	0.027	0.027	<0.001
European	Women	ApoB	MVMR2-IVW	260	0.95	0.84 to 1.07	0.386		
European	Women	ApoB	MVMR2-Egger	260	0.93	0.81 to 1.07	0.299	0.549	<0.001
European	Men	ApoB	IVW	78	0.91	0.83 to 1.00	0.046		
European	Men	ApoB	Weighted median	78	0.95	0.87 to 1.05	0.302		
European	Men	ApoB	MR Egger	78	0.92	0.81 to 1.04	0.185	0.827	
European	Men	ApoB	Conmix	78	0.94	0.88 to 1.00	0.084		
European	Men	ApoB	MVMR1-IVW	134	0.91	0.81 to 1.03	0.138		
European	Men	ApoB	MVMR1-Egger	134	0.85	0.74 to 0.97	0.016	0.028	<0.001
European	Men	ApoB	MVMR2-IVW	214	0.93	0.84 to 1.04	0.209		
European	Men	ApoB	MVMR2-Egger	214	0.92	0.81 to 1.03	0.153	0.479	<0.001
East Asian	Women	ApoB	IVW	82	1.05	0.96 to 1.16	0.279		
East Asian	Women	ApoB	Weighted median	82	1.11	0.99 to 1.24	0.079		

East Asian	Women	ApoB	MR Egger	82	1.05	0.90 to 1.23	0.515	0.976	
East Asian	Women	ApoB	Conmix	82	1.10	1.02 to 1.18	0.018		
East Asian	Women	ApoB	MVMR1-IVW	138	1.02	0.83 to 1.24	0.859		
East Asian	Women	ApoB	MVMR1-Egger	138	1.00	0.79 to 1.27	0.987	0.815	<0.001
East Asian	Women	ApoB	MVMR2-IVW	208	1.05	0.88 to 1.26	0.603		
East Asian	Women	ApoB	MVMR2-Egger	208	1.06	0.86 to 1.30	0.596	0.876	<0.001
East Asian	Men	ApoB	IVW	57	0.97	0.88 to 1.07	0.529		
East Asian	Men	ApoB	Weighted median	57	1.08	0.98 to 1.18	0.134		
East Asian	Men	ApoB	MR Egger	57	1.00	0.88 to 1.13	0.998	0.462	
East Asian	Men	ApoB	Conmix	57	0.83	0.73 to 0.93	0.042		
East Asian	Men	ApoB	MVMR1-IVW	100	0.99	0.88 to 1.11	0.813		
East Asian	Men	ApoB	MVMR1-Egger	100	0.95	0.84 to 1.09	0.471	0.305	<0.001
East Asian	Men	ApoB	MVMR2-IVW	171	0.97	0.87 to 1.09	0.621		
East Asian	Men	ApoB	MVMR2-Egger	171	0.96	0.85 to 1.09	0.565	0.761	<0.001
European	Women	TG	IVW	140	1.44	1.26 to 1.65	<0.001		
European	Women	TG	Weighted median	140	1.15	1.02 to 1.29	0.023		
European	Women	TG	MR Egger	140	1.06	0.86 to 1.31	0.568	<0.001	
European	Women	TG	Conmix	140	1.13	1.01 to 1.34	0.029		
European	Women	TG	MVMR1-IVW	189	1.48	1.29 to 1.70	<0.001		
European	Women	TG	MVMR1-Egger	189	1.24	1.03 to 1.48	0.021	0.003	<0.001
European	Women	TG	MVMR2-IVW	260	1.47	1.29 to 1.67	<0.001		
European	Women	TG	MVMR2-Egger	260	1.32	1.13 to 1.55	0.001	0.027	<0.001

European	Men	TG	IVW	93	1.10	0.97 to 1.26	0.150		
European	Men	TG	Weighted median	93	1.05	0.94 to 1.16	0.406		
European	Men	TG	MR Egger	93	0.85	0.69 to 1.04	0.105	0.001	
European	Men	TG	Conmix	93	1.02	0.94 to 1.14	0.601		
European	Men	TG	MVMR1-IVW	134	1.15	1.01 to 1.31	0.034		
European	Men	TG	MVMR1-Egger	134	1.10	0.93 to 1.31	0.264	0.442	<0.001
European	Men	TG	MVMR2-IVW	214	1.15	1.02 to 1.30	0.024		
European	Men	TG	MVMR2-Egger	214	1.10	0.95 to 1.27	0.216	0.266	<0.001
East Asian	Women	TG	IVW	110	1.24	1.07 to 1.44	0.005		
East Asian	Women	TG	Weighted median	110	1.05	0.95 to 1.15	0.353		
East Asian	Women	TG	MR Egger	110	0.98	0.79 to 1.23	0.875	0.006	
East Asian	Women	TG	Conmix	110	1.12	1.02 to 1.19	0.007		
East Asian	Women	TG	MVMR1-IVW	138	1.20	1.02 to 1.40	0.026		
East Asian	Women	TG	MVMR1-Egger	138	1.06	0.87 to 1.28	0.563	0.030	<0.001
East Asian	Women	TG	MVMR2-IVW	208	1.18	1.02 to 1.36	0.024		
East Asian	Women	TG	MVMR2-Egger	208	1.07	0.91 to 1.26	0.413	0.024	<0.001
East Asian	Men	TG	IVW	69	1.04	0.95 to 1.13	0.446		
East Asian	Men	TG	Weighted median	69	0.97	0.89 to 1.07	0.585		
East Asian	Men	TG	MR Egger	69	0.98	0.86 to 1.11	0.733	0.220	
East Asian	Men	TG	Conmix	69	0.93	0.87 to 1.02	0.100		
East Asian	Men	TG	MVMR1-IVW	100	1.03	0.93 to 1.13	0.591		
East Asian	Men	TG	MVMR1-Egger	100	1.04	0.92 to 1.18	0.503	0.687	<0.001

East Asian	Men	TG	MVMR2-IVW	171	1.03	0.93 to 1.14	0.604		
East Asian	Men	TG	MVMR2-Egger	171	1.03	0.91 to 1.16	0.622	0.922	<0.001
European	Women	Lp(a)	IVW	13	1.01	0.93 to 1.10	0.780		
European	Women	Lp(a)	Weighted median	13	1.02	0.94 to 1.11	0.668		
European	Women	Lp(a)	MR Egger	13	1.09	0.97 to 1.23	0.151	0.106	
European	Women	Lp(a)	Conmix	13	1.03	0.96 to 1.09	0.429		
European	Women	Lp(a)	MVMR1-IVW	189	1.03	0.89 to 1.18	0.705		
European	Women	Lp(a)	MVMR1-Egger	189	1.06	0.92 to 1.22	0.433	0.030	<0.001
European	Women	Lp(a)	MVMR2-IVW	260	1.06	0.93 to 1.21	0.408		
European	Women	Lp(a)	MVMR2-Egger	260	1.08	0.94 to 1.23	0.259	0.104	<0.001
European	Men	Lp(a)	IVW	9	1.01	0.95 to 1.07	0.772		
European	Men	Lp(a)	Weighted median	9	1.02	0.95 to 1.10	0.517		
European	Men	Lp(a)	MR Egger	9	0.98	0.89 to 1.08	0.673	0.453	
European	Men	Lp(a)	Conmix	9	1.01	0.96 to 1.08	0.842		
European	Men	Lp(a)	MVMR1-IVW	134	1.01	0.90 to 1.14	0.851		
European	Men	Lp(a)	MVMR1-Egger	134	1.04	0.92 to 1.18	0.508	0.018	<0.001
European	Men	Lp(a)	MVMR2-IVW	214	1.02	0.91 to 1.14	0.775		
European	Men	Lp(a)	MVMR2-Egger	214	1.03	0.92 to 1.16	0.573	0.146	<0.001
East Asian	Women	Lp(a)	IVW	6	1.03	0.96 to 1.11	0.349		
East Asian	Women	Lp(a)	Weighted median	6	1.04	0.96 to 1.11	0.344		
East Asian	Women	Lp(a)	MR Egger	6	1.05	0.93 to 1.18	0.415	0.741	
East Asian	Women	Lp(a)	Conmix	6	1.03	0.97 to 1.10	0.380		

East Asian	Women	Lp(a)	MVMR1-IVW	138	1.04	0.88 to 1.24	0.645		
East Asian	Women	Lp(a)	MVMR1-Egger	138	1.06	0.89 to 1.26	0.519	0.220	<0.001
East Asian	Women	Lp(a)	MVMR2-IVW	208	1.05	0.90 to 1.23	0.528		
East Asian	Women	Lp(a)	MVMR2-Egger	208	1.08	0.92 to 1.26	0.356	0.066	<0.001
East Asian	Men	Lp(a)	IVW	4	1.01	0.91 to 1.12	0.831		
East Asian	Men	Lp(a)	Weighted median	4	0.98	0.93 to 1.04	0.550		
East Asian	Men	Lp(a)	MR Egger	4	0.88	0.74 to 1.05	0.168	0.089	
East Asian	Men	Lp(a)	Conmix	4	0.98	0.93 to 1.04	0.460		
East Asian	Men	Lp(a)	MVMR1-IVW	100	1.01	0.93 to 1.09	0.860		
East Asian	Men	Lp(a)	MVMR1-Egger	100	1.02	0.95 to 1.10	0.546	0.031	<0.001
East Asian	Men	Lp(a)	MVMR2-IVW	171	1.01	0.93 to 1.09	0.843		
East Asian	Men	Lp(a)	MVMR2-Egger	171	1.02	0.94 to 1.10	0.671	0.221	<0.001

a. ApoB, apolipoprotein B; CI, confidence interval; Conmix, contamination mixture method; IVW, inverse variance weighted; Lp(a), lipoprotein(a); MVMR, multivariable MR; OR, odds ratio; TG, triglycerides.

b. MVMR1 includes apoB, TG and Lp(a); MVMR2 includes apoB, TG, Lp(a) and body mass index.

c. Estimates are expressed in standard deviation for lipid fractions, and in odds ratio for type 2 diabetes.

Additional file 1: Table S5. Mendelian randomization estimates for sex-specific associations of genetically predicted lipid traits (instrumented by the SNPs from the UK Biobank) with glycemic traits in people of European ancestry (including SNPs explaining more of the variance in the outcome than in the exposure).

Sex	Exposure	Outcome	Method	SNPs	Beta	95% CI	*P* value	*P* value (Egger intercept)	*P* value (Q-statistics)
Women	ApoB	HbA1c	IVW	111	0.03	-0.03 to 0.08	0.371		
Women	ApoB	HbA1c	Weighted median	111	-0.02	-0.04 to 0.01	0.198		
Women	ApoB	HbA1c	MR Egger	111	-0.04	-0.12 to 0.03	0.272	0.017	
Women	ApoB	HbA1c	Conmix	111	-0.02	-0.03 to -0.00	0.065		
Women	ApoB	HbA1c	MVMR1-IVW	195	0.01	-0.04 to 0.06	0.750		
Women	ApoB	HbA1c	MVMR1-Egger	195	-0.05	-0.10 to 0.01	0.103	<0.001	<0.001
Women	ApoB	HbA1c	MVMR2-IVW	268	0.01	-0.03 to 0.06	0.630		
Women	ApoB	HbA1c	MVMR2-Egger	268	-0.01	-0.06 to 0.04	0.804	0.108	<0.001
Men	ApoB	HbA1c	IVW	79	0.00	-0.06 to 0.06	0.928		
Men	ApoB	HbA1c	Weighted median	79	-0.01	-0.04 to 0.01	0.283		
Men	ApoB	HbA1c	MR Egger	79	-0.04	-0.12 to 0.04	0.281	0.131	
Men	ApoB	HbA1c	Conmix	79	-0.01	-0.02 to -0.00	0.080		
Men	ApoB	HbA1c	MVMR1-IVW	137	-0.01	-0.06 to 0.05	0.769		
Men	ApoB	HbA1c	MVMR1-Egger	137	-0.06	-0.12 to 0.00	0.055	0.001	<0.001
Men	ApoB	HbA1c	MVMR2-IVW	218	0.00	-0.05 to 0.05	0.930		
Men	ApoB	HbA1c	MVMR2-Egger	218	-0.02	-0.07 to 0.03	0.431	0.071	<0.001
Women	ApoB	Fasting insulin	IVW	71	-0.01	-0.05 to 0.02	0.452		
Women	ApoB	Fasting insulin	Weighted median	71	-0.04	-0.08 to 0.00	0.063		

Women	ApoB	Fasting insulin	MR Egger	71	-0.08	-0.13 to -0.02	0.009	0.007	
Women	ApoB	Fasting insulin	Conmix	71	-0.03	-0.06 to -0.00	0.074		
Women	ApoB	Fasting insulin	MVMR1-IVW	119	-0.04	-0.08 to 0.00	0.066		
Women	ApoB	Fasting insulin	MVMR1-Egger	119	-0.08	-0.13 to -0.02	0.003	0.015	<0.001
Women	ApoB	Fasting insulin	MVMR2-IVW	175	-0.03	-0.07 to 0.01	0.152		
Women	ApoB	Fasting insulin	MVMR2-Egger	175	-0.05	-0.10 to -0.01	0.020	0.025	<0.001
Men	ApoB	Fasting insulin	IVW	54	-0.02	-0.04 to 0.01	0.212		
Men	ApoB	Fasting insulin	Weighted median	54	-0.02	-0.06 to 0.02	0.310		
Men	ApoB	Fasting insulin	MR Egger	54	-0.02	-0.06 to 0.01	0.238	0.721	
Men	ApoB	Fasting insulin	Conmix	54	-0.01	-0.04 to 0.01	0.502		
Men	ApoB	Fasting insulin	MVMR1-IVW	83	0.00	-0.04 to 0.03	0.801		
Men	ApoB	Fasting insulin	MVMR1-Egger	83	-0.02	-0.07 to 0.02	0.275	0.096	<0.001
Men	ApoB	Fasting insulin	MVMR2-IVW	144	0.00	-0.04 to 0.04	0.986		
Men	ApoB	Fasting insulin	MVMR2-Egger	144	0.00	-0.04 to 0.04	0.915	0.818	<0.001
Women	ApoB	Fasting glucose	IVW	71	-0.03	-0.06 to 0.01	0.109		
Women	ApoB	Fasting glucose	Weighted median	71	-0.04	-0.08 to -0.01	0.025		
Women	ApoB	Fasting glucose	MR Egger	71	-0.05	-0.11 to 0.00	0.074	0.309	
Women	ApoB	Fasting glucose	Conmix	71	-0.03	-0.06 to 0.00	0.118		
Women	ApoB	Fasting glucose	MVMR1-IVW	119	-0.02	-0.06 to 0.02	0.235		
Women	ApoB	Fasting glucose	MVMR1-Egger	119	-0.05	-0.10 to -0.00	0.038	0.058	<0.001
Women	ApoB	Fasting glucose	MVMR2-IVW	175	-0.02	-0.06 to 0.02	0.257		
Women	ApoB	Fasting glucose	MVMR2-Egger	175	-0.04	-0.08 to -0.00	0.045	0.032	<0.001

Men	ApoB	Fasting glucose	IVW	54	-0.01	-0.04 to 0.03	0.671		
Men	ApoB	Fasting glucose	Weighted median	54	-0.01	-0.04 to 0.03	0.692		
Men	ApoB	Fasting glucose	MR Egger	54	-0.02	-0.06 to 0.02	0.341	0.329	
Men	ApoB	Fasting glucose	Conmix	54	-0.01	-0.02 to 0.02	0.540		
Men	ApoB	Fasting glucose	MVMR1-IVW	83	0.01	-0.02 to 0.04	0.572		
Men	ApoB	Fasting glucose	MVMR1-Egger	83	-0.01	-0.05 to 0.02	0.418	0.013	<0.001
Men	ApoB	Fasting glucose	MVMR2-IVW	145	0.01	-0.02 to 0.04	0.571		
Men	ApoB	Fasting glucose	MVMR2-Egger	145	0.01	-0.03 to 0.04	0.711	0.711	<0.001
Women	TG	HbA1c	IVW	143	0.07	0.02 to 0.12	0.011		
Women	TG	HbA1c	Weighted median	143	0.00	-0.03 to 0.04	0.874		
Women	TG	HbA1c	MR Egger	143	-0.08	-0.16 to 0.00	0.055	<0.001	
Women	TG	HbA1c	Conmix	143	0.15	0.12 to 0.18	<0.001		
Women	TG	HbA1c	MVMR1-IVW	195	0.06	0.01 to 0.12	0.016		
Women	TG	HbA1c	MVMR1-Egger	195	-0.01	-0.08 to 0.05	0.685	0.001	<0.001
Women	TG	HbA1c	MVMR2-IVW	268	0.06	0.01 to 0.10	0.016		
Women	TG	HbA1c	MVMR2-Egger	268	0.01	-0.05 to 0.07	0.748	0.005	<0.001
Men	TG	HbA1c	IVW	95	-0.03	-0.09 to 0.03	0.295		
Men	TG	HbA1c	Weighted median	95	-0.05	-0.09 to -0.01	0.009		
Men	TG	HbA1c	MR Egger	95	-0.17	-0.26 to -0.09	<0.001	<0.001	
Men	TG	HbA1c	Conmix	95	-0.01	-0.05 to 0.28	0.414		
Men	TG	HbA1c	MVMR1-IVW	137	-0.01	-0.07 to 0.05	0.695		
Men	TG	HbA1c	MVMR1-Egger	137	-0.04	-0.12 to 0.03	0.270	0.219	<0.001

Men	TG	HbA1c	MVMR2-IVW	218	-0.01	-0.07 to 0.04	0.620		
Men	TG	HbA1c	MVMR2-Egger	218	-0.05	-0.11 to 0.01	0.131	0.054	<0.001
Women	TG	Fasting insulin	IVW	93	0.09	0.05 to 0.13	<0.001		
Women	TG	Fasting insulin	Weighted median	93	0.08	0.03 to 0.13	0.001		
Women	TG	Fasting insulin	MR Egger	93	0.01	-0.06 to 0.08	0.748	0.005	
Women	TG	Fasting insulin	Conmix	93	0.10	0.07 to 0.16	<0.001		
Women	TG	Fasting insulin	MVMR1-IVW	119	0.08	0.04 to 0.13	<0.001		
Women	TG	Fasting insulin	MVMR1-Egger	119	0.04	-0.02 to 0.10	0.220	0.025	<0.001
Women	TG	Fasting insulin	MVMR2-IVW	175	0.07	0.03 to 0.12	0.001		
Women	TG	Fasting insulin	MVMR2-Egger	175	0.04	-0.01 to 0.09	0.134	0.056	<0.001
Men	TG	Fasting insulin	IVW	56	0.03	-0.02 to 0.08	0.200		
Men	TG	Fasting insulin	Weighted median	56	0.03	-0.02 to 0.07	0.299		
Men	TG	Fasting insulin	MR Egger	56	-0.05	-0.12 to 0.01	0.129	0.002	
Men	TG	Fasting insulin	Conmix	56	0.02	-0.01 to 0.06	0.178		
Men	TG	Fasting insulin	MVMR1-IVW	83	0.03	-0.01 to 0.07	0.187		
Men	TG	Fasting insulin	MVMR1-Egger	83	-0.02	-0.08 to 0.04	0.461	0.007	<0.001
Men	TG	Fasting insulin	MVMR2-IVW	144	0.02	-0.02 to 0.07	0.280		
Men	TG	Fasting insulin	MVMR2-Egger	144	0.00	-0.05 to 0.05	0.924	0.078	<0.001
Women	TG	Fasting glucose	IVW	93	0.02	-0.01 to 0.05	0.157		
Women	TG	Fasting glucose	Weighted median	93	0.04	0.00 to 0.07	0.042		
Women	TG	Fasting glucose	MR Egger	93	0.00	-0.06 to 0.05	0.896	0.275	
Women	TG	Fasting glucose	Conmix	93	0.04	0.02 to 0.06	0.009		

Women	TG	Fasting glucose	MVMR1-IVW	119	0.01	-0.03 to 0.05	0.582		
Women	TG	Fasting glucose	MVMR1-Egger	119	-0.02	-0.08 to 0.03	0.414	0.070	<0.001
Women	TG	Fasting glucose	MVMR2-IVW	175	0.01	-0.03 to 0.05	0.597		
Women	TG	Fasting glucose	MVMR2-Egger	175	-0.01	-0.05 to 0.04	0.778	0.221	<0.001
Men	TG	Fasting glucose	IVW	56	-0.03	-0.07 to 0.00	0.087		
Men	TG	Fasting glucose	Weighted median	56	0.01	-0.03 to 0.05	0.524		
Men	TG	Fasting glucose	MR Egger	56	-0.04	-0.10 to 0.01	0.141	0.615	
Men	TG	Fasting glucose	Conmix	56	-0.01	-0.03 to 0.03	0.828		
Men	TG	Fasting glucose	MVMR1-IVW	83	-0.02	-0.06 to 0.01	0.197		
Men	TG	Fasting glucose	MVMR1-Egger	83	-0.03	-0.08 to 0.02	0.267	0.822	<0.001
Men	TG	Fasting glucose	MVMR2-IVW	145	-0.03	-0.06 to 0.01	0.162		
Men	TG	Fasting glucose	MVMR2-Egger	145	-0.04	-0.08 to 0.01	0.083	0.305	<0.001
Women	Lp(a)	HbA1c	IVW	15	0.03	0.01 to 0.05	0.012		
Women	Lp(a)	HbA1c	Weighted median	15	0.02	-0.00 to 0.04	0.069		
Women	Lp(a)	HbA1c	MR Egger	15	0.00	-0.03 to 0.03	0.883	0.012	
Women	Lp(a)	HbA1c	Conmix	15	0.02	0.01 to 0.05	0.103		
Women	Lp(a)	HbA1c	MVMR1-IVW	195	0.02	-0.03 to 0.07	0.490		
Women	Lp(a)	HbA1c	MVMR1-Egger	195	0.03	-0.02 to 0.08	0.267	0.024	<0.001
Women	Lp(a)	HbA1c	MVMR2-IVW	268	0.02	-0.02 to 0.07	0.359		
Women	Lp(a)	HbA1c	MVMR2-Egger	268	0.03	-0.02 to 0.08	0.208	0.066	<0.001
Men	Lp(a)	HbA1c	IVW	10	0.02	0.00 to 0.04	0.013		
Men	Lp(a)	HbA1c	Weighted median	10	0.01	-0.01 to 0.03	0.214		

Men	Lp(a)	HbA1c	MR Egger	10	0.00	-0.02 to 0.03	0.744	0.085	
Men	Lp(a)	HbA1c	Conmix	10	0.01	0.00 to 0.02	0.129		
Men	Lp(a)	HbA1c	MVMR1-IVW	137	0.02	-0.03 to 0.08	0.405		
Men	Lp(a)	HbA1c	MVMR1-Egger	137	0.03	-0.02 to 0.09	0.236	0.077	<0.001
Men	Lp(a)	HbA1c	MVMR2-IVW	218	0.02	-0.03 to 0.07	0.353		
Men	Lp(a)	HbA1c	MVMR2-Egger	218	0.03	-0.02 to 0.08	0.227	0.136	<0.001
Women	Lp(a)	Fasting insulin	IVW	4	-0.02	-0.08 to 0.05	0.573		
Women	Lp(a)	Fasting insulin	Weighted median	4	-0.03	-0.10 to 0.04	0.411		
Women	Lp(a)	Fasting insulin	MR Egger	4	-0.01	-0.09 to 0.08	0.883	0.688	
Women	Lp(a)	Fasting insulin	Conmix	4	-0.08	-0.21 to 0.09	0.474		
Women	Lp(a)	Fasting insulin	MVMR1-IVW	119	-0.01	-0.10 to 0.08	0.774		
Women	Lp(a)	Fasting insulin	MVMR1-Egger	119	-0.01	-0.10 to 0.08	0.850	0.733	<0.001
Women	Lp(a)	Fasting insulin	MVMR2-IVW	175	-0.01	-0.09 to 0.08	0.887		
Women	Lp(a)	Fasting insulin	MVMR2-Egger	175	0.00	-0.09 to 0.09	0.922	0.890	<0.001
Men	Lp(a)	Fasting insulin	IVW	2	-0.06	-0.13 to 0.01	0.093		
Men	Lp(a)	Fasting insulin	MVMR1-IVW	83	-0.07	-0.16 to 0.03	0.177		
Men	Lp(a)	Fasting insulin	MVMR1-Egger	83	-0.06	-0.15 to 0.04	0.263	0.458	<0.001
Men	Lp(a)	Fasting insulin	MVMR2-IVW	144	-0.06	-0.15 to 0.03	0.169		
Men	Lp(a)	Fasting insulin	MVMR2-Egger	144	-0.05	-0.14 to 0.04	0.303	0.336	<0.001
Women	Lp(a)	Fasting glucose	IVW	4	0.05	0.00 to 0.11	0.042		
Women	Lp(a)	Fasting glucose	Weighted median	4	0.06	0.01 to 0.12	0.027		
Women	Lp(a)	Fasting glucose	MR Egger	4	0.06	-0.01 to 0.13	0.083	0.752	

Women	Lp(a)	Fasting glucose	Conmix	4	0.06	-0.06 to 0.14	0.087		
Women	Lp(a)	Fasting glucose	MVMR1-IVW	119	0.03	-0.05 to 0.11	0.442		
Women	Lp(a)	Fasting glucose	MVMR1-Egger	119	0.06	-0.02 to 0.14	0.169	0.013	<0.001
Women	Lp(a)	Fasting glucose	MVMR2-IVW	175	0.04	-0.03 to 0.12	0.246		
Women	Lp(a)	Fasting glucose	MVMR2-Egger	175	0.05	-0.02 to 0.13	0.180	0.406	<0.001
Men	Lp(a)	Fasting glucose	IVW	2	-0.01	-0.07 to 0.04	0.687		
Men	Lp(a)	Fasting glucose	MVMR1-IVW	83	-0.04	-0.11 to 0.04	0.344		
Men	Lp(a)	Fasting glucose	MVMR1-Egger	83	-0.03	-0.11 to 0.05	0.424	0.651	<0.001
Men	Lp(a)	Fasting glucose	MVMR2-IVW	145	-0.03	-0.10 to 0.04	0.411		
Men	Lp(a)	Fasting glucose	MVMR2-Egger	145	-0.03	-0.10 to 0.04	0.422	0.946	<0.001

a. ApoB, apolipoprotein B; CI, confidence interval; Conmix, contamination mixture method; IVW, inverse variance weighted; Lp(a), lipoprotein(a); MVMR, multivariable MR; TG, triglycerides.

b. MVMR1 includes apoB, TG and Lp(a); MVMR2 includes apoB, TG, Lp(a) and body mass index.

c. Estimates are expressed in standard deviation for lipid fractions and HbA1c, in pmol/L (natural log transformed) for fasting insulin, and in mmol/L for fasting glucose.

Additional file 1: Table S6. Mendelian randomization estimates for ancestry- and sex-specific associations of genetically predicted lipid traits (instrumented by the SNPs from the UK Biobank) with type 2 diabetes (excluding SNPs explaining more of the variance in the outcome than in the exposure).

Ancestry	Sex	Exposure	Method	SNPs	OR	95% CI	*P* value	*P* value (Egger intercept)	*P* value (Q-statistics)
European	Women	ApoB	IVW	108	0.99	0.90 to 1.09	0.863		
European	Women	ApoB	Weighted median	108	0.91	0.83 to 1.00	0.063		
European	Women	ApoB	MR Egger	108	0.90	0.79 to 1.03	0.110	0.041	
European	Women	ApoB	Conmix	108	0.95	0.89 to 1.03	0.252		
European	Women	ApoB	MVMR1-IVW	187	0.93	0.82 to 1.04	0.193		
European	Women	ApoB	MVMR1-Egger	187	0.82	0.72 to 0.94	0.004	<0.001	<0.001
European	Women	ApoB	MVMR2-IVW	258	0.95	0.85 to 1.07	0.407		
European	Women	ApoB	MVMR2-Egger	258	0.92	0.82 to 1.04	0.183	0.170	<0.001
European	Men	ApoB	IVW	78	0.91	0.83 to 1.00	0.046		
European	Men	ApoB	Weighted median	78	0.95	0.87 to 1.05	0.302		
European	Men	ApoB	MR Egger	78	0.92	0.81 to 1.04	0.185	0.827	
European	Men	ApoB	Conmix	78	0.94	0.88 to 1.00	0.084		
European	Men	ApoB	MVMR1-IVW	133	0.91	0.82 to 1.02	0.097		
European	Men	ApoB	MVMR1-Egger	133	0.84	0.74 to 0.95	0.007	0.014	<0.001
European	Men	ApoB	MVMR2-IVW	213	0.93	0.84 to 1.03	0.175		
European	Men	ApoB	MVMR2-Egger	213	0.92	0.82 to 1.02	0.116	0.405	<0.001
East Asian	Women	ApoB	IVW	82	1.05	0.96 to 1.16	0.279		
East Asian	Women	ApoB	Weighted median	82	1.11	0.99 to 1.24	0.079		

East Asian	Women	ApoB	MR Egger	82	1.05	0.90 to 1.23	0.515	0.976	
East Asian	Women	ApoB	Conmix	82	1.10	1.02 to 1.18	0.018		
East Asian	Women	ApoB	MVMR1-IVW	137	1.04	0.91 to 1.20	0.559		
East Asian	Women	ApoB	MVMR1-Egger	137	0.92	0.78 to 1.09	0.358	0.012	<0.001
East Asian	Women	ApoB	MVMR2-IVW	207	1.08	0.94 to 1.24	0.280		
East Asian	Women	ApoB	MVMR2-Egger	207	1.02	0.88 to 1.20	0.763	0.171	<0.001
East Asian	Men	ApoB	IVW	57	0.97	0.88 to 1.07	0.529		
East Asian	Men	ApoB	Weighted median	57	1.08	0.98 to 1.18	0.134		
East Asian	Men	ApoB	MR Egger	57	1.00	0.88 to 1.13	0.998	0.462	
East Asian	Men	ApoB	Conmix	57	0.83	0.73 to 0.93	0.042		
East Asian	Men	ApoB	MVMR1-IVW	100	0.99	0.88 to 1.11	0.813		
East Asian	Men	ApoB	MVMR1-Egger	100	0.95	0.84 to 1.09	0.471	0.305	<0.001
East Asian	Men	ApoB	MVMR2-IVW	171	0.97	0.87 to 1.09	0.621		
East Asian	Men	ApoB	MVMR2-Egger	171	0.96	0.85 to 1.09	0.565	0.761	<0.001
European	Women	TG	IVW	138	1.40	1.25 to 1.58	<0.001		
European	Women	TG	Weighted median	138	1.15	1.02 to 1.29	0.022		
European	Women	TG	MR Egger	138	1.07	0.89 to 1.29	0.462	<0.001	
European	Women	TG	Conmix	138	1.14	1.03 to 1.30	0.018		
European	Women	TG	MVMR1-IVW	187	1.44	1.27 to 1.63	<0.001		
European	Women	TG	MVMR1-Egger	187	1.23	1.04 to 1.44	0.013	0.004	<0.001
European	Women	TG	MVMR2-IVW	258	1.42	1.27 to 1.60	<0.001		
European	Women	TG	MVMR2-Egger	258	1.31	1.13 to 1.51	<0.001	0.050	<0.001

European	Men	TG	IVW	92	1.09	0.96 to 1.23	0.178		
European	Men	TG	Weighted median	92	1.04	0.94 to 1.16	0.427		
European	Men	TG	MR Egger	92	0.83	0.69 to 1.00	0.044	<0.001	
European	Men	TG	Conmix	92	1.00	0.93 to 1.13	0.967		
European	Men	TG	MVMR1-IVW	133	1.13	1.01 to 1.28	0.041		
European	Men	TG	MVMR1-Egger	133	1.08	0.92 to 1.26	0.341	0.350	<0.001
European	Men	TG	MVMR2-IVW	213	1.13	1.01 to 1.26	0.039		
European	Men	TG	MVMR2-Egger	213	1.08	0.94 to 1.24	0.263	0.296	<0.001
East Asian	Women	TG	IVW	109	1.20	1.08 to 1.34	0.001		
East Asian	Women	TG	Weighted median	109	1.05	0.95 to 1.15	0.349		
East Asian	Women	TG	MR Egger	109	1.03	0.88 to 1.21	0.692	0.011	
East Asian	Women	TG	Conmix	109	1.10	1.02 to 1.18	0.017		
East Asian	Women	TG	MVMR1-IVW	137	1.15	1.03 to 1.29	0.016		
East Asian	Women	TG	MVMR1-Egger	137	1.07	0.93 to 1.23	0.334	0.080	<0.001
East Asian	Women	TG	MVMR2-IVW	207	1.13	1.01 to 1.26	0.032		
East Asian	Women	TG	MVMR2-Egger	207	1.07	0.94 to 1.21	0.295	0.108	<0.001
East Asian	Men	TG	IVW	69	1.04	0.95 to 1.13	0.446		
East Asian	Men	TG	Weighted median	69	0.97	0.89 to 1.07	0.585		
East Asian	Men	TG	MR Egger	69	0.98	0.86 to 1.11	0.733	0.220	
East Asian	Men	TG	Conmix	69	0.93	0.87 to 1.02	0.100		
East Asian	Men	TG	MVMR1-IVW	100	1.03	0.93 to 1.13	0.591		
East Asian	Men	TG	MVMR1-Egger	100	1.04	0.92 to 1.18	0.503	0.687	<0.001

East Asian	Men	TG	MVMR2-IVW	171	1.03	0.93 to 1.14	0.604		
East Asian	Men	TG	MVMR2-Egger	171	1.03	0.91 to 1.16	0.622	0.922	<0.001
European	Women	Lp(a)	IVW	13	1.01	0.93 to 1.10	0.780		
European	Women	Lp(a)	Weighted median	13	1.02	0.94 to 1.11	0.668		
European	Women	Lp(a)	MR Egger	13	1.09	0.97 to 1.23	0.151	0.106	
European	Women	Lp(a)	Conmix	13	1.03	0.96 to 1.09	0.429		
European	Women	Lp(a)	MVMR1-IVW	187	1.03	0.90 to 1.17	0.682		
European	Women	Lp(a)	MVMR1-Egger	187	1.05	0.93 to 1.20	0.427	0.041	<0.001
European	Women	Lp(a)	MVMR2-IVW	258	1.06	0.94 to 1.19	0.370		
European	Women	Lp(a)	MVMR2-Egger	258	1.08	0.95 to 1.21	0.239	0.131	<0.001
European	Men	Lp(a)	IVW	9	1.01	0.95 to 1.07	0.772		
European	Men	Lp(a)	Weighted median	9	1.02	0.95 to 1.10	0.517		
European	Men	Lp(a)	MR Egger	9	0.98	0.89 to 1.08	0.673	0.453	
European	Men	Lp(a)	Conmix	9	1.01	0.96 to 1.08	0.842		
European	Men	Lp(a)	MVMR1-IVW	133	1.01	0.90 to 1.13	0.844		
European	Men	Lp(a)	MVMR1-Egger	133	1.04	0.93 to 1.16	0.489	0.014	<0.001
European	Men	Lp(a)	MVMR2-IVW	213	1.02	0.92 to 1.13	0.771		
European	Men	Lp(a)	MVMR2-Egger	213	1.03	0.93 to 1.15	0.567	0.141	<0.001
East Asian	Women	Lp(a)	IVW	6	1.03	0.96 to 1.11	0.349		
East Asian	Women	Lp(a)	Weighted median	6	1.04	0.96 to 1.11	0.344		
East Asian	Women	Lp(a)	MR Egger	6	1.05	0.93 to 1.18	0.415	0.741	
East Asian	Women	Lp(a)	Conmix	6	1.03	0.97 to 1.10	0.380		

East Asian	Women	Lp(a)	MVMR1-IVW	137	1.04	0.92 to 1.18	0.555		
East Asian	Women	Lp(a)	MVMR1-Egger	137	1.04	0.92 to 1.18	0.506	0.593	<0.001
East Asian	Women	Lp(a)	MVMR2-IVW	207	1.05	0.93 to 1.18	0.447		
East Asian	Women	Lp(a)	MVMR2-Egger	207	1.06	0.94 to 1.20	0.323	0.147	<0.001
East Asian	Men	Lp(a)	IVW	4	1.01	0.91 to 1.12	0.831		
East Asian	Men	Lp(a)	Weighted median	4	0.98	0.93 to 1.04	0.550		
East Asian	Men	Lp(a)	MR Egger	4	0.88	0.74 to 1.05	0.168	0.089	
East Asian	Men	Lp(a)	Conmix	4	0.98	0.93 to 1.04	0.460		
East Asian	Men	Lp(a)	MVMR1-IVW	100	1.01	0.93 to 1.09	0.860		
East Asian	Men	Lp(a)	MVMR1-Egger	100	1.02	0.95 to 1.10	0.546	0.031	<0.001
East Asian	Men	Lp(a)	MVMR2-IVW	171	1.01	0.93 to 1.09	0.843		
East Asian	Men	Lp(a)	MVMR2-Egger	171	1.02	0.94 to 1.10	0.671	0.221	<0.001

a. ApoB, apolipoprotein B; CI, confidence interval; Conmix, contamination mixture method; IVW, inverse variance weighted; Lp(a), lipoprotein(a); MVMR, multivariable MR; OR, odds ratio; TG, triglycerides.

b. MVMR1 includes apoB, TG and Lp(a); MVMR2 includes apoB, TG, Lp(a) and body mass index.

c. The SNPs explaining more of the variance in type 2 diabetes than in the exposure identified by Steiger filtering are rs10440833 and rs76895963 for TG in European women, rs76895963 for TG in European men, and rs10440833 for TG in East Asian women.

d. Estimates are expressed in standard deviation for lipid fractions, and in odds ratio for type 2 diabetes.

Additional file 1: Table S7. Mendelian randomization estimates for sex-specific associations of genetically predicted lipid traits (instrumented by the SNPs from the UK Biobank) with glycemic traits in people of European ancestry (excluding SNPs explaining more of the variance in the outcome than in the exposure).

Sex	Exposure	Outcome	Method	SNPs	Beta	95% CI	*P* value	*P* value (Egger intercept)	*P* value (Q-statistics)
Women	ApoB	HbA1c	IVW	108	0.01	-0.03 to 0.05	0.715		
Women	ApoB	HbA1c	Weighted median	108	-0.02	-0.04 to 0.01	0.156		
Women	ApoB	HbA1c	MR Egger	108	-0.04	-0.10 to 0.02	0.208	0.036	
Women	ApoB	HbA1c	Conmix	108	-0.02	-0.03 to -0.00	0.050		
Women	ApoB	HbA1c	MVMR1-IVW	190	0.00	-0.04 to 0.04	0.945		
Women	ApoB	HbA1c	MVMR1-Egger	190	-0.06	-0.10 to -0.01	0.016	<0.001	<0.001
Women	ApoB	HbA1c	MVMR2-IVW	263	0.01	-0.03 to 0.04	0.794		
Women	ApoB	HbA1c	MVMR2-Egger	263	-0.01	-0.06 to 0.03	0.514	0.035	<0.001
Men	ApoB	HbA1c	IVW	76	-0.02	-0.06 to 0.02	0.247		
Men	ApoB	HbA1c	Weighted median	76	-0.02	-0.04 to 0.01	0.229		
Men	ApoB	HbA1c	MR Egger	76	-0.04	-0.09 to 0.01	0.136	0.345	
Men	ApoB	HbA1c	Conmix	76	-0.01	-0.03 to -0.00	0.110		
Men	ApoB	HbA1c	MVMR1-IVW	135	-0.02	-0.06 to 0.03	0.416		
Men	ApoB	HbA1c	MVMR1-Egger	135	-0.06	-0.11 to -0.01	0.011	<0.001	<0.001
Men	ApoB	HbA1c	MVMR2-IVW	216	-0.01	-0.05 to 0.03	0.589		
Men	ApoB	HbA1c	MVMR2-Egger	216	-0.03	-0.07 to 0.02	0.212	0.054	<0.001
Women	ApoB	Fasting insulin	IVW	71	-0.01	-0.05 to 0.02	0.452		
Women	ApoB	Fasting insulin	Weighted median	71	-0.04	-0.08 to 0.00	0.063		

Women	ApoB	Fasting insulin	MR Egger	71	-0.08	-0.13 to -0.02	0.009	0.007	
Women	ApoB	Fasting insulin	Conmix	71	-0.03	-0.06 to -0.00	0.074		
Women	ApoB	Fasting insulin	MVMR1-IVW	119	-0.04	-0.08 to 0.00	0.066		
Women	ApoB	Fasting insulin	MVMR1-Egger	119	-0.08	-0.13 to -0.02	0.003	0.015	<0.001
Women	ApoB	Fasting insulin	MVMR2-IVW	175	-0.03	-0.07 to 0.01	0.152		
Women	ApoB	Fasting insulin	MVMR2-Egger	175	-0.05	-0.10 to -0.01	0.020	0.025	<0.001
Men	ApoB	Fasting insulin	IVW	54	-0.02	-0.04 to 0.01	0.212		
Men	ApoB	Fasting insulin	Weighted median	54	-0.02	-0.06 to 0.02	0.310		
Men	ApoB	Fasting insulin	MR Egger	54	-0.02	-0.06 to 0.01	0.238	0.721	
Men	ApoB	Fasting insulin	Conmix	54	-0.01	-0.04 to 0.01	0.502		
Men	ApoB	Fasting insulin	MVMR1-IVW	83	0.00	-0.04 to 0.03	0.801		
Men	ApoB	Fasting insulin	MVMR1-Egger	83	-0.02	-0.07 to 0.02	0.275	0.096	<0.001
Men	ApoB	Fasting insulin	MVMR2-IVW	144	0.00	-0.04 to 0.04	0.986		
Men	ApoB	Fasting insulin	MVMR2-Egger	144	0.00	-0.04 to 0.04	0.915	0.818	<0.001
Women	ApoB	Fasting glucose	IVW	71	-0.03	-0.06 to 0.01	0.109		
Women	ApoB	Fasting glucose	Weighted median	71	-0.04	-0.08 to -0.01	0.025		
Women	ApoB	Fasting glucose	MR Egger	71	-0.05	-0.11 to 0.00	0.074	0.309	
Women	ApoB	Fasting glucose	Conmix	71	-0.03	-0.06 to 0.00	0.118		
Women	ApoB	Fasting glucose	MVMR1-IVW	119	-0.02	-0.06 to 0.02	0.235		
Women	ApoB	Fasting glucose	MVMR1-Egger	119	-0.05	-0.10 to -0.00	0.038	0.058	<0.001
Women	ApoB	Fasting glucose	MVMR2-IVW	175	-0.02	-0.06 to 0.02	0.257		
Women	ApoB	Fasting glucose	MVMR2-Egger	175	-0.04	-0.08 to -0.00	0.045	0.032	<0.001

Men	ApoB	Fasting glucose	IVW	54	-0.01	-0.04 to 0.03	0.671		
Men	ApoB	Fasting glucose	Weighted median	54	-0.01	-0.04 to 0.03	0.692		
Men	ApoB	Fasting glucose	MR Egger	54	-0.02	-0.06 to 0.02	0.341	0.329	
Men	ApoB	Fasting glucose	Conmix	54	-0.01	-0.02 to 0.02	0.540		
Men	ApoB	Fasting glucose	MVMR1-IVW	83	0.01	-0.02 to 0.04	0.572		
Men	ApoB	Fasting glucose	MVMR1-Egger	83	-0.01	-0.05 to 0.02	0.418	0.013	<0.001
Men	ApoB	Fasting glucose	MVMR2-IVW	145	0.01	-0.02 to 0.04	0.571		
Men	ApoB	Fasting glucose	MVMR2-Egger	145	0.01	-0.03 to 0.04	0.711	0.711	<0.001
Women	TG	HbA1c	IVW	138	0.06	0.02 to 0.10	0.004		
Women	TG	HbA1c	Weighted median	138	0.00	-0.03 to 0.04	0.879		
Women	TG	HbA1c	MR Egger	138	-0.08	-0.15 to -0.02	0.008	<0.001	
Women	TG	HbA1c	Conmix	138	0.17	0.13 to 0.20	<0.001		
Women	TG	HbA1c	MVMR1-IVW	190	0.06	0.01 to 0.10	0.012		
Women	TG	HbA1c	MVMR1-Egger	190	-0.02	-0.08 to 0.03	0.401	<0.001	<0.001
Women	TG	HbA1c	MVMR2-IVW	263	0.05	0.01 to 0.09	0.016		
Women	TG	HbA1c	MVMR2-Egger	263	0.00	-0.05 to 0.05	0.936	0.001	<0.001
Men	TG	HbA1c	IVW	94	-0.04	-0.09 to 0.01	0.128		
Men	TG	HbA1c	Weighted median	94	-0.05	-0.09 to -0.01	0.007		
Men	TG	HbA1c	MR Egger	94	-0.19	-0.26 to -0.11	<0.001	<0.001	
Men	TG	HbA1c	Conmix	94	-0.01	-0.05 to 0.32	0.308		
Men	TG	HbA1c	MVMR1-IVW	135	-0.02	-0.07 to 0.03	0.532		
Men	TG	HbA1c	MVMR1-Egger	135	-0.06	-0.13 to 0.00	0.054	0.027	<0.001

Men	TG	HbA1c	MVMR2-IVW	216	-0.02	-0.07 to 0.03	0.389		
Men	TG	HbA1c	MVMR2-Egger	216	-0.06	-0.12 to -0.01	0.030	0.011	<0.001
Women	TG	Fasting insulin	IVW	93	0.09	0.05 to 0.13	<0.001		
Women	TG	Fasting insulin	Weighted median	93	0.08	0.03 to 0.13	0.001		
Women	TG	Fasting insulin	MR Egger	93	0.01	-0.06 to 0.08	0.748	0.005	
Women	TG	Fasting insulin	Conmix	93	0.10	0.07 to 0.16	<0.001		
Women	TG	Fasting insulin	MVMR1-IVW	119	0.08	0.04 to 0.13	<0.001		
Women	TG	Fasting insulin	MVMR1-Egger	119	0.04	-0.02 to 0.10	0.220	0.025	<0.001
Women	TG	Fasting insulin	MVMR2-IVW	175	0.07	0.03 to 0.12	0.001		
Women	TG	Fasting insulin	MVMR2-Egger	175	0.04	-0.01 to 0.09	0.134	0.056	<0.001
Men	TG	Fasting insulin	IVW	56	0.03	-0.02 to 0.08	0.200		
Men	TG	Fasting insulin	Weighted median	56	0.03	-0.02 to 0.07	0.299		
Men	TG	Fasting insulin	MR Egger	56	-0.05	-0.12 to 0.01	0.129	0.002	
Men	TG	Fasting insulin	Conmix	56	0.02	-0.01 to 0.06	0.178		
Men	TG	Fasting insulin	MVMR1-IVW	83	0.03	-0.01 to 0.07	0.187		
Men	TG	Fasting insulin	MVMR1-Egger	83	-0.02	-0.08 to 0.04	0.461	0.007	<0.001
Men	TG	Fasting insulin	MVMR2-IVW	144	0.02	-0.02 to 0.07	0.280		
Men	TG	Fasting insulin	MVMR2-Egger	144	0.00	-0.05 to 0.05	0.924	0.078	<0.001
Women	TG	Fasting glucose	IVW	93	0.02	-0.01 to 0.05	0.157		
Women	TG	Fasting glucose	Weighted median	93	0.04	0.00 to 0.07	0.042		
Women	TG	Fasting glucose	MR Egger	93	0.00	-0.06 to 0.05	0.896	0.275	
Women	TG	Fasting glucose	Conmix	93	0.04	0.02 to 0.06	0.009		

Women	TG	Fasting glucose	MVMR1-IVW	119	0.01	-0.03 to 0.05	0.582		
Women	TG	Fasting glucose	MVMR1-Egger	119	-0.02	-0.08 to 0.03	0.414	0.070	<0.001
Women	TG	Fasting glucose	MVMR2-IVW	175	0.01	-0.03 to 0.05	0.597		
Women	TG	Fasting glucose	MVMR2-Egger	175	-0.01	-0.05 to 0.04	0.778	0.221	<0.001
Men	TG	Fasting glucose	IVW	56	-0.03	-0.07 to 0.00	0.087		
Men	TG	Fasting glucose	Weighted median	56	0.01	-0.03 to 0.05	0.524		
Men	TG	Fasting glucose	MR Egger	56	-0.04	-0.10 to 0.01	0.141	0.615	
Men	TG	Fasting glucose	Conmix	56	-0.01	-0.03 to 0.03	0.828		
Men	TG	Fasting glucose	MVMR1-IVW	83	-0.02	-0.06 to 0.01	0.197		
Men	TG	Fasting glucose	MVMR1-Egger	83	-0.03	-0.08 to 0.02	0.267	0.822	<0.001
Men	TG	Fasting glucose	MVMR2-IVW	145	-0.03	-0.06 to 0.01	0.162		
Men	TG	Fasting glucose	MVMR2-Egger	145	-0.04	-0.08 to 0.01	0.083	0.305	<0.001
Women	Lp(a)	HbA1c	IVW	15	0.03	0.01 to 0.05	0.012		
Women	Lp(a)	HbA1c	Weighted median	15	0.02	-0.00 to 0.04	0.069		
Women	Lp(a)	HbA1c	MR Egger	15	0.00	-0.03 to 0.03	0.883	0.012	
Women	Lp(a)	HbA1c	Conmix	15	0.02	0.01 to 0.05	0.103		
Women	Lp(a)	HbA1c	MVMR1-IVW	190	0.02	-0.03 to 0.06	0.409		
Women	Lp(a)	HbA1c	MVMR1-Egger	190	0.03	-0.01 to 0.08	0.165	0.003	<0.001
Women	Lp(a)	HbA1c	MVMR2-IVW	263	0.02	-0.02 to 0.06	0.275		
Women	Lp(a)	HbA1c	MVMR2-Egger	263	0.03	-0.01 to 0.07	0.119	0.013	<0.001
Men	Lp(a)	HbA1c	IVW	10	0.02	0.00 to 0.04	0.013		
Men	Lp(a)	HbA1c	Weighted median	10	0.01	-0.01 to 0.03	0.214		

Men	Lp(a)	HbA1c	MR Egger	10	0.00	-0.02 to 0.03	0.744	0.085	
Men	Lp(a)	HbA1c	Conmix	10	0.01	0.00 to 0.02	0.129		
Men	Lp(a)	HbA1c	MVMR1-IVW	135	0.02	-0.02 to 0.07	0.278		
Men	Lp(a)	HbA1c	MVMR1-Egger	135	0.03	-0.01 to 0.08	0.174	0.152	<0.001
Men	Lp(a)	HbA1c	MVMR2-IVW	216	0.02	-0.02 to 0.06	0.244		
Men	Lp(a)	HbA1c	MVMR2-Egger	216	0.03	-0.01 to 0.07	0.169	0.234	<0.001
Women	Lp(a)	Fasting insulin	IVW	4	-0.02	-0.08 to 0.05	0.573		
Women	Lp(a)	Fasting insulin	Weighted median	4	-0.03	-0.10 to 0.04	0.411		
Women	Lp(a)	Fasting insulin	MR Egger	4	-0.01	-0.09 to 0.08	0.883	0.688	
Women	Lp(a)	Fasting insulin	Conmix	4	-0.08	-0.21 to 0.09	0.474		
Women	Lp(a)	Fasting insulin	MVMR1-IVW	119	-0.01	-0.10 to 0.08	0.774		
Women	Lp(a)	Fasting insulin	MVMR1-Egger	119	-0.01	-0.10 to 0.08	0.850	0.733	<0.001
Women	Lp(a)	Fasting insulin	MVMR2-IVW	175	-0.01	-0.09 to 0.08	0.887		
Women	Lp(a)	Fasting insulin	MVMR2-Egger	175	0.00	-0.09 to 0.09	0.922	0.890	<0.001
Men	Lp(a)	Fasting insulin	IVW	2	-0.06	-0.13 to 0.01	0.093		
Men	Lp(a)	Fasting insulin	MVMR1-IVW	83	-0.07	-0.16 to 0.03	0.177		
Men	Lp(a)	Fasting insulin	MVMR1-Egger	83	-0.06	-0.15 to 0.04	0.263	0.458	<0.001
Men	Lp(a)	Fasting insulin	MVMR2-IVW	144	-0.06	-0.15 to 0.03	0.169		
Men	Lp(a)	Fasting insulin	MVMR2-Egger	144	-0.05	-0.14 to 0.04	0.303	0.336	<0.001
Women	Lp(a)	Fasting glucose	IVW	4	0.05	0.00 to 0.11	0.042		
Women	Lp(a)	Fasting glucose	Weighted median	4	0.06	0.01 to 0.12	0.027		
Women	Lp(a)	Fasting glucose	MR Egger	4	0.06	-0.01 to 0.13	0.083	0.752	

Women	Lp(a)	Fasting glucose	Conmix	4	0.06	-0.06 to 0.14	0.087		
Women	Lp(a)	Fasting glucose	MVMR1-IVW	119	0.03	-0.05 to 0.11	0.442		
Women	Lp(a)	Fasting glucose	MVMR1-Egger	119	0.06	-0.02 to 0.14	0.169	0.013	<0.001
Women	Lp(a)	Fasting glucose	MVMR2-IVW	175	0.04	-0.03 to 0.12	0.246		
Women	Lp(a)	Fasting glucose	MVMR2-Egger	175	0.05	-0.02 to 0.13	0.180	0.406	<0.001
Men	Lp(a)	Fasting glucose	IVW	2	-0.01	-0.07 to 0.04	0.687		
Men	Lp(a)	Fasting glucose	MVMR1-IVW	83	-0.04	-0.11 to 0.04	0.344		
Men	Lp(a)	Fasting glucose	MVMR1-Egger	83	-0.03	-0.11 to 0.05	0.424	0.651	<0.001
Men	Lp(a)	Fasting glucose	MVMR2-IVW	145	-0.03	-0.10 to 0.04	0.411		
Men	Lp(a)	Fasting glucose	MVMR2-Egger	145	-0.03	-0.10 to 0.04	0.422	0.946	<0.001

a. ApoB, apolipoprotein B; CI, confidence interval; Conmix, contamination mixture method; IVW, inverse variance weighted; Lp(a), lipoprotein(a); MVMR, multivariable MR; TG, triglycerides.

b. MVMR1 includes apoB, TG and Lp(a); MVMR2 includes apoB, TG, Lp(a) and body mass index.

c. The SNPs explaining more of the variance in the outcome than in the exposure identified by Steiger filtering are rs1800562, rs597808 and rs7140110 for apoB on HbA1c in women, rs2519093, rs6602911 and rs80215559 for apoB on HbA1c in men, rs10440833, rs12486657, rs2519093, rs7140110 and rs76895963 for TG on HbA1c in women, and rs76895963 for TG on HbA1c in men.

d. Estimates are expressed in standard deviation for lipid fractions and HbA1c, in pmol/L (natural log transformed) for fasting insulin, and in mmol/L for fasting glucose.

Additional file 1: Table S8. Mendelian randomization estimates for sex-specific associations of genetically predicted lipid traits (instrumented by the SNPs from the UK Biobank) with sex hormones in people of European ancestry (including SNPs explaining more of the variance in the outcome than in the exposure).

Sex	Exposure	Outcome	Method	SNPs	Beta	95% CI	*P* value	*P* value (Egger intercept)	*P* value (Q-statistics)
Women	ApoB	Testosterone	IVW	111	-0.02	-0.06 to 0.01	0.250		
Women	ApoB	Testosterone	Weighted median	111	-0.01	-0.03 to 0.01	0.357		
Women	ApoB	Testosterone	MR Egger	111	0.00	-0.05 to 0.05	0.961	0.245	
Women	ApoB	Testosterone	Conmix	111	-0.02	-0.04 to 0.00	0.115		
Women	ApoB	Testosterone	MVMR1-IVW	195	0.02	-0.02 to 0.05	0.395		
Women	ApoB	Testosterone	MVMR1-Egger	195	0.01	-0.03 to 0.06	0.570	0.748	<0.001
Women	ApoB	Testosterone	MVMR2-IVW	268	0.02	-0.01 to 0.05	0.186		
Women	ApoB	Testosterone	MVMR2-Egger	268	0.02	-0.02 to 0.06	0.300	0.710	<0.001
Men	ApoB	Testosterone	IVW	79	0.00	-0.02 to 0.03	0.691		
Men	ApoB	Testosterone	Weighted median	79	0.00	-0.02 to 0.02	0.830		
Men	ApoB	Testosterone	MR Egger	79	0.01	-0.02 to 0.04	0.629	0.777	
Men	ApoB	Testosterone	Conmix	79	0.01	-0.00 to 0.03	0.286		
Men	ApoB	Testosterone	MVMR1-IVW	137	0.00	-0.03 to 0.02	0.950		
Men	ApoB	Testosterone	MVMR1-Egger	137	0.00	-0.03 to 0.03	0.812	0.720	<0.001
Men	ApoB	Testosterone	MVMR2-IVW	218	-0.01	-0.03 to 0.02	0.634		
Men	ApoB	Testosterone	MVMR2-Egger	218	-0.02	-0.04 to 0.01	0.242	0.063	<0.001
Women	ApoB	Estradiol	IVW	101	0.05	-0.02 to 0.11	0.148		
Women	ApoB	Estradiol	Weighted median	101	0.08	-0.02 to 0.17	0.114		

Women	ApoB	Estradiol	MR Egger	101	0.11	0.02 to 0.20	0.012	0.039	
Women	ApoB	Estradiol	Conmix	101	0.09	0.01 to 0.15	0.040		
Women	ApoB	Estradiol	MVMR1-IVW	177	0.05	-0.02 to 0.11	0.142		
Women	ApoB	Estradiol	MVMR1-Egger	177	0.07	-0.00 to 0.14	0.061	0.236	0.355
Women	ApoB	Estradiol	MVMR2-IVW	244	0.05	-0.02 to 0.11	0.137		
Women	ApoB	Estradiol	MVMR2-Egger	244	0.06	-0.01 to 0.13	0.114	0.575	0.148
Men	ApoB	Estradiol	IVW	73	-0.10	-0.20 to 0.00	0.054		
Men	ApoB	Estradiol	Weighted median	73	-0.07	-0.17 to 0.04	0.209		
Men	ApoB	Estradiol	MR Egger	73	-0.02	-0.16 to 0.11	0.742	0.077	
Men	ApoB	Estradiol	Conmix	73	-0.11	-0.19 to -0.00	0.057		
Men	ApoB	Estradiol	MVMR1-IVW	123	-0.08	-0.17 to 0.02	0.104		
Men	ApoB	Estradiol	MVMR1-Egger	123	-0.06	-0.17 to 0.05	0.282	0.554	<0.001
Men	ApoB	Estradiol	MVMR2-IVW	196	-0.07	-0.16 to 0.01	0.099		
Men	ApoB	Estradiol	MVMR2-Egger	196	-0.07	-0.16 to 0.03	0.172	0.710	<0.001
Women	TG	Testosterone	IVW	143	-0.10	-0.14 to -0.07	<0.001		
Women	TG	Testosterone	Weighted median	143	-0.06	-0.09 to -0.04	<0.001		
Women	TG	Testosterone	MR Egger	143	-0.11	-0.17 to -0.05	0.001	0.812	
Women	TG	Testosterone	Conmix	143	-0.07	-0.08 to -0.05	<0.001		
Women	TG	Testosterone	MVMR1-IVW	195	-0.12	-0.16 to -0.08	<0.001		
Women	TG	Testosterone	MVMR1-Egger	195	-0.14	-0.20 to -0.09	<0.001	0.281	<0.001
Women	TG	Testosterone	MVMR2-IVW	268	-0.13	-0.17 to -0.10	<0.001		
Women	TG	Testosterone	MVMR2-Egger	268	-0.14	-0.18 to -0.10	<0.001	0.467	<0.001

Men	TG	Testosterone	IVW	95	0.04	0.01 to 0.06	0.012		
Men	TG	Testosterone	Weighted median	95	0.03	0.00 to 0.06	0.036		
Men	TG	Testosterone	MR Egger	95	0.02	-0.02 to 0.06	0.381	0.360	
Men	TG	Testosterone	Conmix	95	0.04	0.01 to 0.05	0.052		
Men	TG	Testosterone	MVMR1-IVW	137	0.04	0.01 to 0.07	0.002		
Men	TG	Testosterone	MVMR1-Egger	137	0.04	0.00 to 0.08	0.028	0.891	<0.001
Men	TG	Testosterone	MVMR2-IVW	218	0.05	0.02 to 0.07	0.001		
Men	TG	Testosterone	MVMR2-Egger	218	0.04	0.00 to 0.07	0.036	0.281	<0.001
Women	TG	Estradiol	IVW	128	-0.09	-0.16 to -0.03	0.004		
Women	TG	Estradiol	Weighted median	128	-0.03	-0.13 to 0.07	0.574		
Women	TG	Estradiol	MR Egger	128	-0.06	-0.16 to 0.04	0.264	0.426	
Women	TG	Estradiol	Conmix	128	-0.11	-0.22 to -0.03	0.015		
Women	TG	Estradiol	MVMR1-IVW	177	-0.10	-0.17 to -0.03	0.003		
Women	TG	Estradiol	MVMR1-Egger	177	-0.06	-0.15 to 0.03	0.212	0.154	0.355
Women	TG	Estradiol	MVMR2-IVW	244	-0.10	-0.17 to -0.03	0.004		
Women	TG	Estradiol	MVMR2-Egger	244	-0.06	-0.14 to 0.03	0.172	0.116	0.148
Men	TG	Estradiol	IVW	84	-0.24	-0.33 to -0.16	<0.001		
Men	TG	Estradiol	Weighted median	84	-0.18	-0.29 to -0.08	0.001		
Men	TG	Estradiol	MR Egger	84	-0.14	-0.27 to -0.01	0.035	0.044	
Men	TG	Estradiol	Conmix	84	-0.23	-0.35 to -0.14	<0.001		
Men	TG	Estradiol	MVMR1-IVW	123	-0.24	-0.35 to -0.14	<0.001		
Men	TG	Estradiol	MVMR1-Egger	123	-0.17	-0.31 to -0.03	0.015	0.101	<0.001

Men	TG	Estradiol	MVMR2-IVW	196	-0.22	-0.31 to -0.12	<0.001		
Men	TG	Estradiol	MVMR2-Egger	196	-0.21	-0.33 to -0.09	0.001	0.909	<0.001
Women	Lp(a)	Testosterone	IVW	15	0.00	-0.01 to 0.02	0.920		
Women	Lp(a)	Testosterone	Weighted median	15	0.00	-0.02 to 0.02	0.882		
Women	Lp(a)	Testosterone	MR Egger	15	0.00	-0.03 to 0.02	0.859	0.762	
Women	Lp(a)	Testosterone	Conmix	15	0.00	-0.01 to 0.02	0.530		
Women	Lp(a)	Testosterone	MVMR1-IVW	195	0.00	-0.04 to 0.04	0.843		
Women	Lp(a)	Testosterone	MVMR1-Egger	195	-0.01	-0.05 to 0.03	0.539	0.026	<0.001
Women	Lp(a)	Testosterone	MVMR2-IVW	268	0.00	-0.04 to 0.03	0.930		
Women	Lp(a)	Testosterone	MVMR2-Egger	268	-0.01	-0.04 to 0.03	0.635	0.044	<0.001
Men	Lp(a)	Testosterone	IVW	10	0.01	-0.00 to 0.03	0.053		
Men	Lp(a)	Testosterone	Weighted median	10	0.02	-0.00 to 0.03	0.095		
Men	Lp(a)	Testosterone	MR Egger	10	0.01	-0.02 to 0.03	0.486	0.538	
Men	Lp(a)	Testosterone	Conmix	10	0.01	-0.00 to 0.03	0.076		
Men	Lp(a)	Testosterone	MVMR1-IVW	137	0.02	-0.01 to 0.04	0.149		
Men	Lp(a)	Testosterone	MVMR1-Egger	137	0.02	-0.01 to 0.04	0.133	0.643	<0.001
Men	Lp(a)	Testosterone	MVMR2-IVW	218	0.02	-0.01 to 0.04	0.131		
Men	Lp(a)	Testosterone	MVMR2-Egger	218	0.02	-0.01 to 0.04	0.146	0.908	<0.001
Women	Lp(a)	Estradiol	IVW	13	0.09	0.03 to 0.15	0.004		
Women	Lp(a)	Estradiol	Weighted median	13	0.10	0.02 to 0.18	0.016		
Women	Lp(a)	Estradiol	MR Egger	13	0.09	-0.01 to 0.19	0.067	0.966	
Women	Lp(a)	Estradiol	Conmix	13	0.10	0.03 to 0.16	0.012		

Women	Lp(a)	Estradiol	MVMR1-IVW	177	0.07	0.01 to 0.13	0.030		
Women	Lp(a)	Estradiol	MVMR1-Egger	177	0.07	0.01 to 0.14	0.026	0.602	0.355
Women	Lp(a)	Estradiol	MVMR2-IVW	244	0.07	0.00 to 0.14	0.035		
Women	Lp(a)	Estradiol	MVMR2-Egger	244	0.07	0.00 to 0.14	0.042	0.885	0.148
Men	Lp(a)	Estradiol	IVW	8	0.02	-0.05 to 0.09	0.621		
Men	Lp(a)	Estradiol	Weighted median	8	0.04	-0.04 to 0.12	0.283		
Men	Lp(a)	Estradiol	MR Egger	8	0.10	-0.01 to 0.20	0.068	0.051	
Men	Lp(a)	Estradiol	Conmix	8	0.04	-0.03 to 0.11	0.317		
Men	Lp(a)	Estradiol	MVMR1-IVW	123	0.02	-0.07 to 0.12	0.622		
Men	Lp(a)	Estradiol	MVMR1-Egger	123	0.02	-0.08 to 0.12	0.661	0.839	<0.001
Men	Lp(a)	Estradiol	MVMR2-IVW	196	0.03	-0.06 to 0.12	0.570		
Men	Lp(a)	Estradiol	MVMR2-Egger	196	0.03	-0.06 to 0.12	0.528	0.701	<0.001

a. ApoB, apolipoprotein B; CI, confidence interval; Conmix, contamination mixture method; IVW, inverse variance weighted; Lp(a), lipoprotein(a); MVMR, multivariable MR; TG, triglycerides.

b. MVMR1 includes apoB, TG and Lp(a); MVMR2 includes apoB, TG, Lp(a) and body mass index.

c. Estimates are expressed in standard deviation for lipid fractions and testosterone, and in log odds ratio (above and below the limit of detection) for estradiol.

Additional file 1: Table S9. Mendelian randomization estimates for sex -specific associations of genetically predicted lipid traits (instrumented by the SNPs from the UK Biobank) with sex hormones in people of European ancestry (excluding SNPs explaining more of the variance in the outcome than in the exposure).

Sex	Exposure	Outcome	Method	SNPs	Beta	95% CI	*P* value	*P* value (Egger intercept)	*P* value (Q-statistics)
Women	ApoB	Testosterone	IVW	110	-0.02	-0.05 to 0.02	0.297		
Women	ApoB	Testosterone	Weighted median	110	-0.01	-0.03 to 0.01	0.354		
Women	ApoB	Testosterone	MR Egger	110	0.00	-0.05 to 0.05	0.933	0.383	
Women	ApoB	Testosterone	Conmix	110	-0.01	-0.04 to 0.00	0.105		
Women	ApoB	Testosterone	MVMR1-IVW	194	0.01	-0.02 to 0.05	0.422		
Women	ApoB	Testosterone	MVMR1-Egger	194	0.01	-0.03 to 0.05	0.751	0.467	<0.001
Women	ApoB	Testosterone	MVMR2-IVW	267	0.02	-0.01 to 0.05	0.200		
Women	ApoB	Testosterone	MVMR2-Egger	267	0.02	-0.02 to 0.05	0.395	0.474	<0.001
Men	ApoB	Testosterone	IVW	79	0.00	-0.02 to 0.03	0.691		
Men	ApoB	Testosterone	Weighted median	79	0.00	-0.02 to 0.02	0.830		
Men	ApoB	Testosterone	MR Egger	79	0.01	-0.02 to 0.04	0.629	0.777	
Men	ApoB	Testosterone	Conmix	79	0.01	-0.00 to 0.03	0.286		
Men	ApoB	Testosterone	MVMR1-IVW	137	0.00	-0.03 to 0.02	0.950		
Men	ApoB	Testosterone	MVMR1-Egger	137	0.00	-0.03 to 0.03	0.812	0.720	<0.001
Men	ApoB	Testosterone	MVMR2-IVW	218	-0.01	-0.03 to 0.02	0.634		
Men	ApoB	Testosterone	MVMR2-Egger	218	-0.02	-0.04 to 0.01	0.242	0.063	<0.001
Women	ApoB	Estradiol	IVW	101	0.05	-0.02 to 0.11	0.148		
Women	ApoB	Estradiol	Weighted median	101	0.08	-0.02 to 0.17	0.114		

Women	ApoB	Estradiol	MR Egger	101	0.11	0.02 to 0.20	0.012	0.039		
Women	ApoB	Estradiol	Conmix	101	0.09	0.01 to 0.15	0.040			
Women	ApoB	Estradiol	MVMR1-IVW	177	0.05	-0.02 to 0.11	0.142			
Women	ApoB	Estradiol	MVMR1-Egger	177	0.07	-0.00 to 0.14	0.061	0.236	0.355	
Women	ApoB	Estradiol	MVMR2-IVW	244	0.05	-0.02 to 0.11	0.137			
Women	ApoB	Estradiol	MVMR2-Egger	244	0.06	-0.01 to 0.13	0.114	0.575	0.148	
Men	ApoB	Estradiol	IVW	73	-0.10	-0.20 to 0.00	0.054			
Men	ApoB	Estradiol	Weighted median	73	-0.07	-0.17 to 0.04	0.209			
Men	ApoB	Estradiol	MR Egger	73	-0.02	-0.16 to 0.11	0.742	0.077		
Men	ApoB	Estradiol	Conmix	73	-0.11	-0.19 to -0.00	0.057			
Men	ApoB	Estradiol	MVMR1-IVW	123	-0.08	-0.17 to 0.02	0.104			
Men	ApoB	Estradiol	MVMR1-Egger	123	-0.06	-0.17 to 0.05	0.282	0.554	<0.001	
Men	ApoB	Estradiol	MVMR2-IVW	196	-0.07	-0.16 to 0.01	0.099			
Men	ApoB	Estradiol	MVMR2-Egger	196	-0.07	-0.16 to 0.03	0.172	0.710	<0.001	
Women	TG	Testosterone	IVW	142	-0.10	-0.13 to -0.06	<0.001			
Women	TG	Testosterone	Weighted median	142	-0.06	-0.09 to -0.04	<0.001			
Women	TG	Testosterone	MR Egger	142	-0.11	-0.17 to -0.05	<0.001	0.524		
Women	TG	Testosterone	Conmix	142	-0.06	-0.08 to -0.05	<0.001			
Women	TG	Testosterone	MVMR1-IVW	194	-0.12	-0.16 to -0.08	<0.001			
Women	TG	Testosterone	MVMR1-Egger	194	-0.14	-0.19 to -0.09	<0.001	0.141	<0.001	
Women	TG	Testosterone	MVMR2-IVW	267	-0.13	-0.16 to -0.09	<0.001			
Women	TG	Testosterone	MVMR2-Egger	267	-0.14	-0.18 to -0.10	<0.001	0.280	<0.001	

Men	TG	Testosterone	IVW	95	0.04	0.01 to 0.06	0.012		
Men	TG	Testosterone	Weighted median	95	0.03	0.00 to 0.06	0.036		
Men	TG	Testosterone	MR Egger	95	0.02	-0.02 to 0.06	0.381	0.360	
Men	TG	Testosterone	Conmix	95	0.04	0.01 to 0.05	0.052		
Men	TG	Testosterone	MVMR1-IVW	137	0.04	0.01 to 0.07	0.002		
Men	TG	Testosterone	MVMR1-Egger	137	0.04	0.00 to 0.08	0.028	0.891	<0.001
Men	TG	Testosterone	MVMR2-IVW	218	0.05	0.02 to 0.07	0.001		
Men	TG	Testosterone	MVMR2-Egger	218	0.04	0.00 to 0.07	0.036	0.281	<0.001
Women	TG	Estradiol	IVW	128	-0.09	-0.16 to -0.03	0.004		
Women	TG	Estradiol	Weighted median	128	-0.03	-0.13 to 0.07	0.574		
Women	TG	Estradiol	MR Egger	128	-0.06	-0.16 to 0.04	0.264	0.426	
Women	TG	Estradiol	Conmix	128	-0.11	-0.22 to -0.03	0.015		
Women	TG	Estradiol	MVMR1-IVW	177	-0.10	-0.17 to -0.03	0.003		
Women	TG	Estradiol	MVMR1-Egger	177	-0.06	-0.15 to 0.03	0.212	0.154	0.355
Women	TG	Estradiol	MVMR2-IVW	244	-0.10	-0.17 to -0.03	0.004		
Women	TG	Estradiol	MVMR2-Egger	244	-0.06	-0.14 to 0.03	0.172	0.116	0.148
Men	TG	Estradiol	IVW	84	-0.24	-0.33 to -0.16	<0.001		
Men	TG	Estradiol	Weighted median	84	-0.18	-0.29 to -0.08	0.001		
Men	TG	Estradiol	MR Egger	84	-0.14	-0.27 to -0.01	0.035	0.044	
Men	TG	Estradiol	Conmix	84	-0.23	-0.35 to -0.14	<0.001		
Men	TG	Estradiol	MVMR1-IVW	123	-0.24	-0.35 to -0.14	<0.001		
Men	TG	Estradiol	MVMR1-Egger	123	-0.17	-0.31 to -0.03	0.015	0.101	<0.001

Men	TG	Estradiol	MVMR2-IVW	196	-0.22	-0.31 to -0.12	<0.001		
Men	TG	Estradiol	MVMR2-Egger	196	-0.21	-0.33 to -0.09	0.001	0.909	<0.001
Women	Lp(a)	Testosterone	IVW	15	0.00	-0.01 to 0.02	0.920		
Women	Lp(a)	Testosterone	Weighted median	15	0.00	-0.02 to 0.02	0.882		
Women	Lp(a)	Testosterone	MR Egger	15	0.00	-0.03 to 0.02	0.859	0.762	
Women	Lp(a)	Testosterone	Conmix	15	0.00	-0.01 to 0.02	0.530		
Women	Lp(a)	Testosterone	MVMR1-IVW	194	0.00	-0.04 to 0.04	0.922		
Women	Lp(a)	Testosterone	MVMR1-Egger	194	-0.01	-0.05 to 0.03	0.531	0.005	<0.001
Women	Lp(a)	Testosterone	MVMR2-IVW	267	0.00	-0.03 to 0.03	0.974		
Women	Lp(a)	Testosterone	MVMR2-Egger	267	-0.01	-0.04 to 0.03	0.647	0.010	<0.001
Men	Lp(a)	Testosterone	IVW	10	0.01	-0.00 to 0.03	0.053		
Men	Lp(a)	Testosterone	Weighted median	10	0.02	-0.00 to 0.03	0.095		
Men	Lp(a)	Testosterone	MR Egger	10	0.01	-0.02 to 0.03	0.486	0.538	
Men	Lp(a)	Testosterone	Conmix	10	0.01	-0.00 to 0.03	0.076		
Men	Lp(a)	Testosterone	MVMR1-IVW	137	0.02	-0.01 to 0.04	0.149		
Men	Lp(a)	Testosterone	MVMR1-Egger	137	0.02	-0.01 to 0.04	0.133	0.643	<0.001
Men	Lp(a)	Testosterone	MVMR2-IVW	218	0.02	-0.01 to 0.04	0.131		
Men	Lp(a)	Testosterone	MVMR2-Egger	218	0.02	-0.01 to 0.04	0.146	0.908	<0.001
Women	Lp(a)	Estradiol	IVW	13	0.09	0.03 to 0.15	0.004		
Women	Lp(a)	Estradiol	Weighted median	13	0.10	0.02 to 0.18	0.016		
Women	Lp(a)	Estradiol	MR Egger	13	0.09	-0.01 to 0.19	0.067	0.966	
Women	Lp(a)	Estradiol	Conmix	13	0.10	0.03 to 0.16	0.012		

Women	Lp(a)	Estradiol	MVMR1-IVW	177	0.07	0.01 to 0.13	0.030		
Women	Lp(a)	Estradiol	MVMR1-Egger	177	0.07	0.01 to 0.14	0.026	0.602	0.355
Women	Lp(a)	Estradiol	MVMR2-IVW	244	0.07	0.00 to 0.14	0.035		
Women	Lp(a)	Estradiol	MVMR2-Egger	244	0.07	0.00 to 0.14	0.042	0.885	0.148
Men	Lp(a)	Estradiol	IVW	8	0.02	-0.05 to 0.09	0.621		
Men	Lp(a)	Estradiol	Weighted median	8	0.04	-0.04 to 0.12	0.283		
Men	Lp(a)	Estradiol	MR Egger	8	0.10	-0.01 to 0.20	0.068	0.051	
Men	Lp(a)	Estradiol	Conmix	8	0.04	-0.03 to 0.11	0.317		
Men	Lp(a)	Estradiol	MVMR1-IVW	123	0.02	-0.07 to 0.12	0.622		
Men	Lp(a)	Estradiol	MVMR1-Egger	123	0.02	-0.08 to 0.12	0.661	0.839	<0.001
Men	Lp(a)	Estradiol	MVMR2-IVW	196	0.03	-0.06 to 0.12	0.570		
Men	Lp(a)	Estradiol	MVMR2-Egger	196	0.03	-0.06 to 0.12	0.528	0.701	<0.001

a. ApoB, apolipoprotein B; CI, confidence interval; Conmix, contamination mixture method; IVW, inverse variance weighted; Lp(a), lipoprotein(a); MVMR, multivariable MR; TG, triglycerides.

b. MVMR1 includes apoB, TG and Lp(a); MVMR2 includes apoB, TG, Lp(a) and body mass index.

c. The SNPs explaining more of the variance in the outcome than in the exposure identified by Steiger filtering are rs13247874 for apoB on testosterone in women, and rs7199293 for TG on testosterone in women.

d. Estimates are expressed in standard deviation for lipid fractions and testosterone, and in log odds ratio (above and below the limit of detection) for estradiol.
